# Efficacy and Safety of Anti-CD38 Monoclonal Antibodies in Patients with Relapsed or Refractory Multiple Myeloma: A Meta-Analysis of Randomized Clinical Trials

**DOI:** 10.3390/jpm14040360

**Published:** 2024-03-29

**Authors:** Francisco Cezar Aquino de Moraes, Vitor Kendi Tsuchiya Sano, Artur de Oliveira Macena Lôbo, Francinny Alves Kelly, Victória Morbach, Eric Pasqualotto, Rommel Mario Rodríguez Burbano

**Affiliations:** 1 Department of Medicine, Federal University of Pará, Belém 66073-005, Brazil; 2 Department of Medicine, Federal University of Acre, Rio Branco 69920-900, Brazil; vitor.sano@sou.ufac.br; 3 Department of Medicine, Federal University of Pernambuco, Recife 50670-901, Brazil; artur.lobo@ufpe.br; 4 Dante Pazzanese Institute of Cardiology, São Paulo 04012-909, Brazil; francinny.kelly@uscsonline.com.br; 5 Department of Medicine, Feevale University, Novo Hamburgo 93510-235, Brazil; 0277614@feevale.br; 6 Department of Medicine, Federal University of Santa Catarina, Florianopolis 88040-900, Brazil; eric.pasqualotto@grad.ufsc.br; 7 Ophir Loyola Hospital, Belém 66063-240, Brazil; rommel@ufpa.br

**Keywords:** multiple myeloma, anti-CD38 monoclonal antibody, proteasome inhibitors, dexamethasone

## Abstract

The benefit of associating anti-CD38 monoclonal antibodies to proteasome inhibitor (PI)/immunomodulatory agent (IA) and dexamethasone in the treatment of patients with relapsed or refractory multiple myeloma (MM) remains unclear. PubMed, Embase, and Cochrane Library databases were searched for randomized controlled trials that investigated the addition of anti-CD38 monoclonal antibodies to a therapy composed of PI/IA and dexamethasone versus PI/IA and dexamethasone alone for treating relapsed or refractory MM. Hazard ratios (HRs) or risk ratios (RRs) were computed for binary endpoints, with 95% confidence intervals (CIs). Six studies comprising 2191 patients were included. Anti-CD38 monoclonal antibody significantly improved progression-free survival (HR 0.52; 95% CI 0.43–0.61; *p* < 0.001) and overall survival (HR 0.72; 95% CI 0.63–0.83; *p* < 0.001). There was a significant increase in hematological adverse events, such as neutropenia (RR 1.41; 95% CI 1.26–1.58; *p* < 0.01) and thrombocytopenia (RR 1.14; 95% CI 1.02–1.27; *p* = 0.02), in the group treated with anti-CD38 monoclonal antibody. Also, there was a significant increase in non-hematological adverse events, such as dyspnea (RR 1.72; 95% CI 1.38–2.13; *p* < 0.01) and pneumonia (RR 1.34; 95% CI 1.13–1.59; *p* < 0.01), in the group treated with anti-CD38 monoclonal antibody. In conclusion, the incorporation of an anti-CD38 monoclonal antibody demonstrated a promising prospect for reshaping the established MM treatment paradigms.

## 1. Introduction

Multiple myeloma (MM) is a neoplasm characterized by the clonal expansion of malignant plasma cells (PCs) in the bone marrow (BM) [1,2,3,4]. The onset of MM occurs with the asymptomatic pre-malignant proliferation of PCs, which comprises monoclonal gammopathy of undetermined significance (MGUS) and smoldering myeloma (SMM) [5,6,7,8,9]. MGUS comprises patients with serum M-protein levels (<3 g/dL) and monoclonal PC in the BM (<10%), while patients with serum M-protein levels (≥3 g/dL) and/or PC in the BM (≥10%) are classified as MM [7,10,11]. In addition, the diagnosis should consider end-organ damage resulting from the serum M-spike and/or monoclonal PC in the BM [12,13].

MM accounts for 1% of all cancers and is the second most common hematologic neoplasm in the world, representing 30,000 new cases per year, with an estimated incidence of 5 cases per 100,000 [14,15,16]. The implementation of emerging therapies has increased the overall survival of patients with MM since the 21st century [17,18,19]. Before 2000, the average survival for MM was only 12 months, whereas after 2000, it increased to 24 months [20,21,22].

MM remains incurable, and most patients undergo several lines of treatment, with the choice relying on exposure and previous response [20,23,24,25]. Currently, clinical treatment options include proteasome inhibitors, immunomodulatory agents, steroids, alkylating agents, and monoclonal antibodies, often combined with autologous stem cell transplantation in eligible patients [12,26,27,28]. The need for new therapeutic approaches for relapsed or refractory MM has generated monoclonal antibodies targeting CD38, including daratumumab and isatuximab [29,30,31,32,33].

Thus, this meta-analysis of phase III randomized controlled trials (RCTs) aimed to clarify the benefit of treatment with anti-CD38 monoclonal antibodies combined with proteasome inhibitors or immunomodulatory agents for patients with relapsed or refractory MM.

## 2. Materials and Methods

### 2.1. Protocol and Registration

This research followed the recommendations outlined by the Preferred Reporting Items for Systematic Reviews and Meta-Analysis (PRISMA) guidelines (Tables S1 and S2) [34]. The protocol was registered in the International Prospective Register of Systematic Reviews (PROSPERO) with the registration number CRD42024507495.

### 2.2. Eligibility Criteria

Included studies must have met the following eligibility criteria: (1) phase III RCTs; (2) enrolling adult patients (≥18 years) with documented relapsed or refractory MM; (3) treatment regimens with dexamethasone and immunomodulatory agent/proteasome inhibitor for intervention and control groups and an anti-CD38 humanized IgG1-κ monoclonal antibody for the intervention group only; and (4) Eastern Cooperative Oncology Group (ECOG) performance status score of 0, 1, or 2. Studies with no outcomes of interest, overlapping populations, or non-randomized clinical trials were excluded. The eligibility criteria for each of the RCTs included in this systematic review and meta-analysis are detailed in Table S3.

The question we sought to answer was as follows: How effective is the addition of anti-CD38 monoclonal antibody to dexamethasone and immunomodulatory agent/proteasome inhibitor therapy for the treatment of patients with relapsed or refractory MM?

### 2.3. Search Strategy

PubMed, Embase, and Cochrane Library were searched on 29 January 2024. The search strategies utilized for each database are summarized in detail in Table S4.

To identify potentially relevant future studies, we checked the references of the included articles and systematic reviews in the literature. Additionally, we set up alerts in each database to notify us of new publications related to the topic of interest. The studies found in the databases and in the references of the articles were incorporated into the reference management software (Rayyan) [35]. Two reviewers (F.A.K. and V.M.S.) selected the studies found in the databases individually. Discordances in selection were solved by a general agreement between three authors (F.A.K., V.M.S., and F.C.A.d.M.).

### 2.4. Data Extraction

To summarize the main findings, two authors (A.d.O.M.L. and V.K.T.S.) independently collected the data extracted from the included articles. The following baseline characteristics were extracted: (1) sample size; (2) age; (3) sex; (4) race; (5) ECOG status; (6) disease stage according to the International Staging System; and (7) type of measurable MM (IgG or non-IgG).

The ensuing outcomes of interest were extracted: (1) progression-free survival (PFS), defined as the time elapsed from patient randomization to the occurrence of death from any cause or disease progression; (2) overall survival (OS), defined as the length of time, counted from the start of treatment, that patients are still alive; and (3) adverse events, defined as an untoward medical occurrence related to a treatment. These were evaluated according to the Common Terminology Criteria for Adverse Events, version 5.0 [36].

### 2.5. Endpoints and Subgroup Analysis

The outcomes of interest were (1) PFS; (2) OS; patients with any grade and, in another analysis, grade ≥ 3 of (3) anemia; (4) febrile neutropenia; (5) lymphopenia; (6) neutropenia; (7) thrombocytopenia; (8) arthralgia; (9) asthenia; (10) back pain; (11) bronchitis; (12) constipation; (13) cough; (14) diarrhea; (15) dyspnea; (16) fatigue; (17) hypertension; (18) insomnia; (19) nausea; (20) peripheral edema; (21) pneumonia; (22) pyrexia; and (23) upper respiratory tract infection.

### 2.6. Risk of Bias Assessment

To assess the quality of individual randomized studies, the Cochrane risk of bias tool for randomized trials (RoB-2) was used [37]. A score of low, high, or unclear risk of bias was assigned to each trial across five domains: (1) randomization process; (2) deviations from intended interventions; (3) missing outcomes; (4) measurement of outcomes; and (5) selection of reported results. To further examine the possibility of publication bias, funnel-plot analyses were employed. Two authors (V.K.T.S. and F.C.A.d.M.) performed an independent evaluation of the risk of bias for all included RCTs and any disagreements were resolved by consensus.

### 2.7. Statistical Analysis

Hazard ratios (HRs) or risk ratios (RRs) were computed for binary outcomes, with 95% confidence intervals (CIs). Cochran Q-test and I^2^ statistics were utilized to evaluate heterogeneity; *p* values < 0.10 and I^2^ > 25% were indicative of a statistically significant heterogeneity between the included RCTs [38]. The Sidik–Jonkman estimator was used to determine the Tau^2^ variance between studies [39]. For all endpoints, DerSimonian and Laird random effect models were used [40]. To assess publication bias, contour-enhanced funnel plots were visually inspected and assessed using Egger’s regression asymmetry [41]. The statistical analyses were executed through the R Software version 4.3.

## 3. Results

### 3.1. Search Results and Characteristics of Included Studies

**Figure 1:**
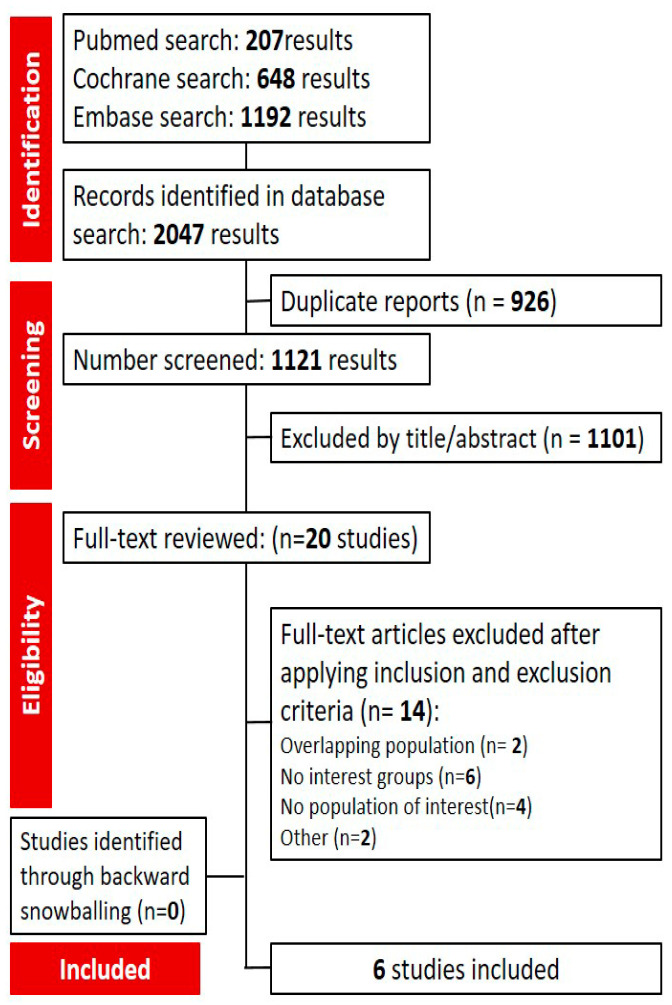
Flow diagram of the articles included in the meta-analysis.

The selection was described in a PRISMA flow diagram (Figure 1). A total of 2047 references were found in the systematic search. After the elimination of identical references and the evaluation according to the content in the title and abstract, 20 studies were deemed fit for the full-text reading, which encompassed a thorough evaluation of the inclusion and exclusion criteria. Out of these, six RCTs were included, comprising a total of 2191 patients [42,43,44,45,46,47].

A total of 1162 patients with relapsed or refractory MM were randomized to receive anti-CD38 monoclonal antibodies and 1029 patients were assigned to the control group. The majority of patients had an ECOG performance status score of 0 (770 patients) and 811 patients had an ECOG ≥ 1. Regarding the type of measurable MM, 979 were IgG positive and 641 were non-IgG. The median age ranged from 28.0 to 90.0 years. Baseline patient and study characteristics are summarized in Table 1 and Table S5.

### 3.2. Results Based on Outcome

#### 3.2.1. Progression-Free Survival

**Figure 2:**
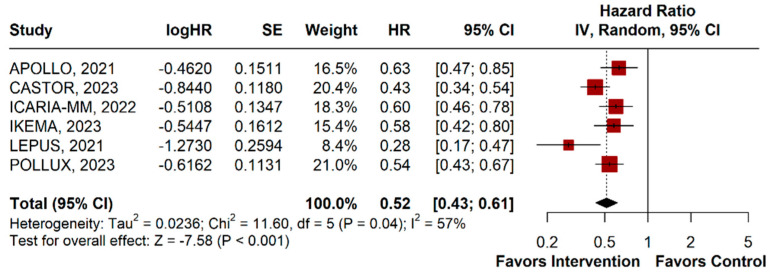
Progression-free survival of patients with multiple myeloma treated with anti-CD38 monoclonal antibodies versus contro [42,43,44,45,46,47].

PFS was evaluated in six RCTs, comprising a total of 2191 patients. Anti-CD38 monoclonal antibodies significantly improved PFS compared to the control group (HR 0.52, 95% CI 0.43–0.61; *p* < 0.001; I^2^ = 57%; Figure 2).

#### 3.2.2. Overall Survival

**Figure 3:**
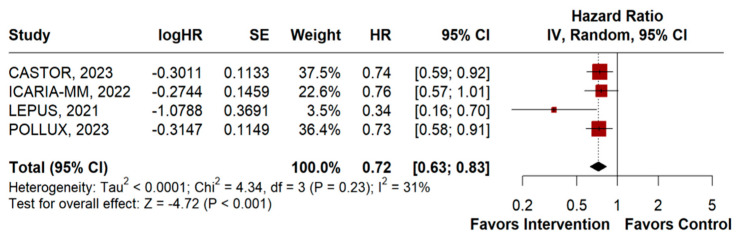
Overall survival of patients with multiple myeloma treated with anti-CD38 monoclonal antibodies versus control [43,45,46,47].

OS was evaluated in four RCTs, comprising a total of 1562 patients. Anti-CD38 monoclonal antibodies significantly improved OS compared to the control group (HR 0.72, 95% CI 0.63–0.83; *p* < 0.001; I^2^ = 31%; Figure 3).

#### 3.2.3. Adverse Events

Anti-CD38 monoclonal antibodies increased any grade of arthralgia (RR 1.69, 95% CI 1.07–2.69; *p* = 0.03; I^2^ = 70%; Figure S1B), back pain (RR 1.38, 95% CI 1.05–1.82; *p* = 0.02; I^2^ = 47%; Figure S1D), bronchitis (RR 1.89, 95% CI 1.30–2.75; *p* < 0.01; I^2^ = 59%; Figure S1E), cough (RR 2.19, 95% CI 1.77–2.70; *p* < 0.01; I^2^ = 0%; Figure S1G), diarrhea (RR 1.41, 95% CI 1.23–1.63; *p* < 0.01; I^2^ = 21%; Figure S1H), dyspnea (RR 1.72, 95% CI 1.38–2.13; *p* < 0.01; I^2^ = 0%; Figure S1I), febrile neutropenia (RR 2.83, 95% CI 1.65–4.87; *p* < 0.01; I^2^ = 0%; Figure S1K), nausea (RR 1.55, 95% CI 1.23–1.95; *p* < 0.01; I^2^ = 0%; Figure S1O), neutropenia (RR 1.41, 95% CI 1.26–1.58; *p* < 0.01; I^2^ = 26%; Figure S1P), peripheral edema (RR 1.70, 95% CI 1.27–2.28; *p* < 0.01; I^2^ = 27%; Figure S1Q), pneumonia (RR 1.34, 95% CI 1.13–1.59; *p* < 0.01; I^2^ = 0%; Figure S1R), pyrexia (RR 1.63, 95% CI 1.33–1.99; *p* < 0.01; I^2^ = 0%; Figure S1S), thrombocytopenia (RR 1.14, 95% CI 1.02–1.27; *p* = 0.02; I^2^ = 50%; Figure S1T), and upper respiratory tract infection (RR 1.64, 95% CI 1.43–1.89; *p* < 0.01; I^2^ = 0%; Figure S1U). 

There was no significant difference between groups in any grade of anemia (RR 0.99, 95% CI 0.90–1.09; *p* = 0.83; I^2^ = 46%; Figure S1A), asthenia (RR 1.00, 95% CI 0.81–1.24; *p* = 0.97; I^2^ = 17%; Figure S1C), constipation (RR 1.01, 95% CI 0.71–1.43; *p* = 0.96; I^2^ = 68%; Figure S1F), fatigue (RR 1.36, 95% CI 0.97–1.91; *p* = 0.08; I^2^ = 76%; Figure S1J), hypertension (RR 2.38, 95% CI 0.81–6.99; *p* = 0.11; I^2^ = 86%; Figure S1L), insomnia (RR 1.20, 95% CI 0.99–1.45; *p* = 0.07; I^2^ = 0%; Figure S1M), and lymphopenia (RR 1.62, 95% CI 0.96–2.74; *p* = 0.07; I^2^ = 73%; Figure S1N). These results are summarized in Table 2.

Anti-CD38 monoclonal antibodies increased grade ≥ 3 of diarrhea (RR 1.95, 95% CI 1.10–3.47; *p* = 0.02; I^2^ = 25%; Figure S2H), dyspnea (RR 5.32, 95% CI 2.39–11.84; *p* < 0.01; I^2^ = 0%; Figure S2I), fatigue (RR 1.86, 95% CI 1.19–2.91; *p* < 0.01; I^2^ = 0%; Figure S2J), febrile neutropenia (RR 2.83, 95% CI 1.65–4.87; *p* < 0.01; I^2^ = 0%; Figure S2K), lymphopenia (RR 2.13, 95% CI 1.24–3.64; *p* < 0.01; I^2^ = 57%; Figure S2N), neutropenia (RR 1.64, 95% CI 1.33–2.01; *p* < 0.01; I^2^ = 58%; Figure S2P), pneumonia (RR 1.31, 95% CI 1.06–1.63; *p* = 0.01; I^2^ = 0%; Figure S2R), thrombocytopenia (RR 1.25, 95% CI 1.08–1.44; *p* < 0.01; I^2^ = 0%; Figure S2T), and upper respiratory tract infection (RR 1.97, 95% CI 1.02–3.79; *p* = 0.04; I^2^ = 7%; Figure S2U). 

However, there was no significant difference in grade ≥ 3 of anemia (RR 1.00, 95% CI 0.81–1.24; *p* = 0.99; I^2^ = 18%; Figure S2A), arthralgia (RR 1.62, 95% CI 0.65–4.04; *p* = 0.30; I^2^ = 0%; Figure S2B), asthenia (RR 1.08, 95% CI 0.52–2.22; *p* = 0.84; I^2^ = 30%; Figure S2C), back pain (RR 1.98, 95% CI 0.97–4.04; *p* = 0.06; I^2^ = 0%; Figure S2D), bronchitis (RR 1.78, 95% CI 0.83–3.84; *p* = 0.14; I^2^ = 15%; Figure S2E), constipation (RR 0.88, 95% CI 0.10–7.90; *p* = 0.91; I^2^ = 43%; Figure S2F), cough (RR 0.52, 95% CI 0.02–14.73; *p* = 0.70; I^2^ = 57%; Figure S2G), hypertension (RR 2.94, 95% CI 0.62–13.96; *p* = 0.18; I^2^ = 84%; Figure S2L), insomnia (RR 1.30, 95% CI 0.62–2.71; *p* = 0.48; I^2^ = 0%; Figure S2M), nausea (RR 3.31, 95% CI 0.81–13.56; *p* = 0.10; I^2^ = 0%; Figure S2O), peripheral edema (RR 1.25, 95% CI 0.36–4.27; *p* = 0.73; I^2^ = 0%; Figure S2Q), and pyrexia (RR 1.47, 95% CI 0.73–2.98; *p* = 0.28; I^2^ = 0%; Figure S2S). These results are summarized in Table 3.

#### 3.2.4. Sensitivity Analysis and Quality Assessment

We executed a leave-one-out sensitivity analysis for PFS and OS outcomes. The outcomes showed stability, without changes in significance with the removal of each individual study. However, there was a significant reduction in heterogeneity among studies with the removal of Lepus et al., from I^2^ = 31% to I^2^ = 0% for OS, and a reduction from I^2^ = 57% to I^2^ = 28% for PFS. The leave-one-out sensitivity analysis plots are detailed in Figure S3. 

**Figure 4:**
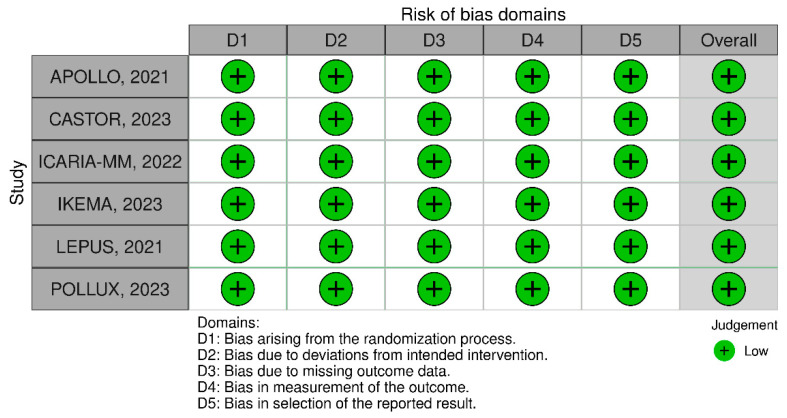
Critical appraisal of randomized controlled trials according to the Cochrane Collaboration tool for assessing risk of bias in randomized trials [42,43,44,45,46,47].

The individual quality assessment of each study included in the meta-analysis is depicted in Figure 4. The six studies included had a low risk of bias in all five domains of Rob 2, which represents a high quality of RCTs included in the analysis. As shown in Figures S4 and S5, the funnel plots of PFS and OS outcomes present a symmetrical distribution of similar-weight studies, indicating no evidence of significant publication bias and a lower variance among the studies included.

## 4. Discussion

In this systematic review and meta-analysis involving six RCTs and 2191 patients, we compared dexamethasone and immunomodulatory agent/proteasome inhibitor for both intervention and control groups and an anti-CD38 humanized IgG1-κ monoclonal antibody intervention for Relapsed or Refractory Multiple Myeloma. The main results of the pooled analyses were as follows: (1) PFS was better in patients in the anti-CD38 group; (2) OS showed a significant difference in favor of the anti-CD38 group; and (3) adverse events grade ≥ 3 such as neutropenia, thrombocytopenia, diarrhea, dyspnea, and pneumonia occurred in a significantly higher proportion of patients in the anti-CD38 group compared to the control.

CD38 is a type II transmembrane protein that is involved in cellular calcium signaling, lymphocyte activation, and the migration of these immune cells [43]. In the normal state, CD38 expression is low, while in MM it is remarkably high in plasma cells [44]. Thus, the use of targeted therapies such as daratumumab is justified due to the inhibition of tumor growth by binding to the CD38 glycoprotein with high affinity, with the activation of immune-mediated molecular mechanisms [47]. Its use in clinical practice is currently approved by the European Medicines Agency (EMA) and the US Food and Drug Administration (FDA) as a standard single treatment or in combination with other anti-tumor therapies for MM [43]. 

In addition, isatuximab is an IgG1 monoclonal antibody responsible for the enzymatic regulation of CD38, and treatment with this agent is justified because it induces caspase-dependent apoptosis and can act synergistically by invigorating T cells and natural killer cells [43]. Although similar to daratumab, the mechanisms of action of these two drugs differ because they have different target epitopes [46]. Furthermore, isatuximab can induce cell death directly, whereas daratumab requires additional antitumor combinations; therefore, its treatment is based on the kinergic effect of the combined protocols [46,47].

The mechanism of action of anti-CD38 monoclonal antibodies is important for the treatment of MM because they act on specific receptors (Fc) for antibodies, which are very expressed in this disease [26,48,49]. Their use for treating MM is justified mainly due to complement-dependent cytotoxicity (CDC), in which the binding of C1q complex to the Fc tail of the therapeutic antibody initiates the complement cascade and causes the generation of the membrane attack complex [50,51,52,53]. This leads to the deposition of complement factors on the membrane, triggering the engulfment and destruction of tumor cells by phagocytes, which is further elevated by the synergy with increased expression of Fcγ receptors [53,54,55].

Other Fc-dependent cellular mechanisms include antibody-dependent cytotoxicity (ADCC) and antibody-dependent cellular phagocytosis (ADCP) [56]. ADCC mainly involves natural killer (NK) cells, which recognize tumor cells and then release cytotoxic granules such as perforin and granzymes, which induce the death of target cells [57,58,59]. On the other hand, ADCP involves the phagocytosis of cells by macrophages, which recognize the target cells by antibodies bound to the surface of the tumor cells; thus, this marking by Fc receptors leads to the destruction of these cells [60,61,62]. The combination of these mechanisms, ADC and ADCP, plays a crucial role in the efficacy of daratumumab and isatuximab, although the latter can also induce cell death by direct mechanisms that are independent of Fc [63,64,65].

Our findings provide compelling evidence that incorporating anti-CD38 therapy into the treatment regimen of multiple myeloma (MM) patients significantly improves PFS. The analysis revealed an HR of 0.5, indicating a 50% reduction in the risk of disease progression for patients receiving anti-CD38 treatment compared to those who did not. This is statistically significant, with a *p*-value of less than 0.01, highlighting the robust nature of this benefit. These results are particularly encouraging when compared to the outcomes of other emerging therapies for MM. In the KarMMa trial, a phase 2 study investigating the efficacy and safety of idecabtagene vicleucel, a chimeric antigen receptor (CAR) T-cell therapy targeting B-cell maturation antigen (BCMA), notable results were observed. The trial enrolled heavily pretreated patients with relapsed and refractory myeloma, who had received at least three prior treatment regimens. Among the 128 patients who received CART-cell, the median progression-free survival was 8.8 months (95% confidence interval, 5.6 to 11.6), indicating a substantial benefit in delaying disease progression. This stands in stark contrast to the substantial PFS benefit observed in our study with anti-CD38 therapy [66].

Our study also revealed a significant improvement in OS for patients treated with anti-CD38 monoclonal antibodies. The analysis yielded an HR of 0.72, indicating a 28% reduction in the mortality risk for patients receiving this therapy compared to the control group. This finding holds strong statistical significance with a *p*-value of less than 0.01, emphasizing the positive impact of anti-CD38 treatment on patient longevity. The observed benefit in OS aligns with the results of the KarMMa trial, which investigated the efficacy and safety of idecabtagene vicleucel (ide-cel, also known as bb2121), a chimeric antigen receptor (CAR) T-cell therapy targeting B-cell maturation antigen (BCMA). In the KarMMa trial, the Kaplan–Meier estimated median overall survival was 19.4 months (95% CI, 18.2 could not be estimated), with an overall survival of 78% at 12 months. These data underscore the promising potential of anti-CD38 therapy in improving overall survival outcomes for patients with refractory and relapsed myeloma [66].

For patients with MM undergoing chemotherapy, treatment-related adverse events can significantly impact their well-being and quality of life, affecting their daily routines and emotional state [67,68]. Our meta-analysis suggests that while anti-CD38 monoclonal antibodies may be associated with an increased risk of severe lymphopenia, neutropenic infections, and thrombocytopenia, they also offer improved clinical efficacy in MM treatment. Notably, other serious non-hematological adverse events include diarrhea, dyspnea, and pneumonia. Nonetheless, considering the potential benefits to clinical outcomes, the further investigation and incorporation of anti-CD38 as a treatment option for MM may be warranted, with careful consideration of both the risks and benefits.

The main limitation of this meta-analysis is the high heterogeneity (I^2^ > 25%) present in most of the analyzed outcomes, such as progression-free survival and overall survival. This suggests that there may be significant differences between the populations included in the studies. However, despite this limitation, this meta-analysis was able to draw robust conclusions demonstrating the significant benefit of anti-CD38 for patients with relapsed or refractory MM.

## 5. Conclusions

This is the first meta-analysis of randomized clinical trials to evaluate the efficacy and safety of anti-CD38 therapy for the treatment of patients with relapsed or refractory multiple myeloma. Our results suggest that this therapy represents a potential treatment option, and its application in clinical practice should be encouraged.

## Figures and Tables

**Table 1:** Design and characteristics of studies included in the meta-analysis.

Study	Designs	Sample Size	Intervention	Age ^†^	Sex Male/Female	Race	ECOG Status, No (%)	Prior Lines of Therapy	International Staging System Disease Stage	Type of Measurable MM IgG/Non-IgG
APOLLO, 2021 [42]	RCT-phase III	IG:151 CG:153	IG: Daratumumab + polamalidomide + dexamethasone CG: Pomalidomide + dexamethasone	IG: 67 (42–86) CG: 68 (35–90)	IG: 79/72 CG: 82/71	IG: White-135 (89%); Non-white-16 (11%) CG: White-137 (90%); Non-white-16 (10%)	IG: 0–91 (60%); ≥1–60 (40%); CG: 0–77 (50%); ≥1–76 (50%)	IG: 1–16 (11%); 2/3–114 (75%); ≥4–21 (14%) CG: 1–18 (12%); 2/3–113 (74%); ≥4–22 (14%)	IG: I-68 (45%); II-50 (33%); III-33 (22%) CG: I-69 (45%); II-51 (33%); III-33 (22%)	IG: IgG-62 (41%); Non-IgG-89 (59%) CG: IgG-63 (41%); Non-IgG-90 (59%)
CASTOR, 2023 [47]	RCT-phase III	IG:251 CG:247	IG: Daratumumab + bortezomib + dexamethasone CG: Bortezomib + dexamethasone	Overall:64 (30–88)	IG:137/114 CG:148/99	IG: White-216 (86%); Non-white-35(14%) CG: White-219 (88%); Non-white-28 (12%)	IG: 0–106 (42%); ≥1–144 (58%); Not reported: 1 CG: 0–116 (47%); ≥1–131 (53%)	IG: 1–122 (48.6%); 2/3–107 (42.6%); ≥4–22 (9.2%) CG: 1–113 (45.7%); 2/3–106 (42.9%); ≥4–28 (11.4%)	IG: I-98 (39%); II-94 (37%); III-59 (24%) CG: I-96 (39%); II-100 (40%); III-42 (21%)	IG: IgG-125 (67%); Non-IgG-61 (33%); Unknown: 65 CG: IgG-138 (70%); Non-IgG-58 (30%); Unknown: 51
ICARIA-MM, 2022 [43]	RCT-phase III	IG:154 CG:153	IG: Isatuximab + pomalidomide + dexamethasone CG: Pomalidomide + dexamethasone	IG:68 CG:66	IG:89/65 CG:70/83	NA	NA	NA	IG: I-64 (42%); II-53 (34%); III-34 (22%); Unknown-3 (2%) CG: I-51 (33%); II-56 (37%); III-26 (14.5%); Unknown-1 (0.6%)	IG: IgG-102 (66%); Non-IgG-52 (34%) CG: IgG-100 (65%); Non-IgG-53 (35%)
IKEMA, 2023 [44]	RCT-phase III	IG:179 CG:123	IG: Isatuximab + carfilzomib + dexamethasone CG: Carfilzomib + dexamethasone	IG:65 (37–86) CG:63 (33–90)	NA	NA	NA	IG: 1–79 (44.1%); 2/3–97 (55.9%); ≥4–0 CG: 1–55 (44.7%); 2/3–66 (55.3%); ≥4–0	IG: I-89 (49.7%); II-63 (35.2%); III-34 (22%); Unknown-3 (2%) CG: I-71 (57.7%); II-31 (25.2%); III-20 (16.3%); Unknown-1 (0.8%)	NA
LEPUS, 2021 [45]	RCT-phase III	IG:141 CG:70	IG: Daratumumab + bortezomib + dexamethasone CG: Bortezomib + dexamethasone	IG:61.0 (28–79) CG:61.0 (43–82)	IG:85/56 CG:42/28	NA	IG: 0–64 (45.4%); ≥1–77 (54.6%); CG: 0–27 (38.6%); ≥1–43 (61.4%);	IG: 1–41 (29.1%); 2/3–70 (49.6%); ≥4–30 (21.3%) CG: 1–19 (27.1%); 2/3–33 (47.1%); ≥4–18 (25.7%)	IG: I-72 (51.1%); II-45 (31.9%); III-24 (17%) CG: I-34 (48.6%); II-22 (31.4%); III-14 (20%)	IG: IgG-52 (36.9%); Non-IgG-89 (63.1%) CG: IgG-28 (40%); Non-IgG-42 (60%)
POLLUX, 2023 [46]	RCT-phase III	IG:286 CG:283	IG: Daratumumab + lenalidomide + dexamethasone CG: lenalidomide + dexamethasone	IG:65.0 (34–89) CG:65.0 (42–87)	IG:173/113 CG:164/119	IG:: White-207 (72.4)%); Non-white-79 (27.6%) CG: White-186 (65.7%); Non-white-97 (34.3%)	IG: 0–139 (48.6%); ≥1–147 (51.4%); CG: 0–150 (53%); ≥1–133 (47%);	IG: 1–149 (52%); 2/3–123 (43%); ≥4–14 (5%) CG: 1–146 (51%); 2/3–118 (41.7%); ≥4–19 (7.3%)	IG: I-137 (48%); II-93 (32.5%); III-56 (19.5%) CG: I-140 (49.5%); II-86 (30.4%); III-57 (20.1%)	G: IgG-151 (73.6%); Non-IgG-54 (26.4%); Unknown: 81 CG: IgG-158 (74.9%); Non-IgG-53 (25.1%); Unknown: 72

^†^ Median (range). CG, control group; ECOG, Eastern Cooperative Oncology Group; IG, interventional group; MM, multiple myeloma; RCT, randomized controlled trial.

**Table 2:** Adverse events of any grade.

Adverse Events	Events/Total Intervention	Events/Total Control	RR	95% CI	*p*-Value
Hematological adverse events					
Anemia	543/1144	434/1007	0.99	0.90–1.09	0.83
Febrile neutropenia	49/584	17/580	2.83	1.60–4.87	<0.01
Lymphopenia	155/815	73/736	1.62	0.96–2.74	0.07
Neutropenia	606/1144	376/1007	1.41	1.26–1.58	<0.01
Thrombocytopenia	607/1144	425/1007	1.14	1.02–1.27	0.02
Non-hematological adverse events					
Arthralgia	180/855	101/789	1.69	1.07–2.69	0.03
Asthenia	181/1004	168/939	1.00	0.81–1.24	0.97
Back pain	205/855	135/789	1.38	1.05–1.82	0.02
Bronchitis	187/855	97/789	1.89	1.30–2.75	<0.01
Constipation	199/818	164/735	1.01	0.71–1.43	0.96
Cough	246/843	98/708	2.19	1.77–2.70	<0.01
Diarrhea	458/1144	281/1007	1.41	1.23–1.63	<0.01
Dyspnea	198/855	102/789	1.72	1.38–2.13	<0.01
Fatigue	303/1004	218/939	1.36	0.97–1.91	0.08
Hypertension	124/560	54/427	2.38	0.81–6.99	0.11
Insomnia	198/843	139/708	1.20	0.99–1.45	0.07
Nausea	147/678	94/667	1.55	1.23–1.95	<0.01
Peripheral edema	151/678	88/667	1.70	1.27–2.28	<0.01
Pneumonia	263/1144	175/1007	1.34	1.13–1.59	<0.01
Pyrexia	222/967	122/885	1.63	1.33–1.99	<0.01
Upper respiratory tract infection	423/1144	224/1007	1.64	1.43–1.89	<0.01

**Table 3:** Adverse events of grade ≥ 3.

Adverse Events	Events/Total Intervention	Events/Total Control	RR	95% CI	*p*-Value
Hematological adverse events					
Anemia	205/1144	173/1007	1.00	0.81–1.24	0.99
Febrile neutropenia	49/584	17/580	2.83	1.65–4.87	<0.01
Lymphopenia	121/815	43/736	2.13	1.24–3.64	<0.01
Neutropenia	455/1144	276/1007	1.64	1.33–2.01	<0.01
Thrombocytopenia	330/1144	225/1007	1.25	1.08–1.44	<0.01
Non-hematological adverse events					
Arthralgia	15/855	7/789	1.62	0.65–4.04	0.30
Asthenia	29/1004	23/939	1.08	0.52–2.22	0.84
Back pain	23/855	11/789	1.98	0.97–4.04	0.06
Bronchitis	27/855	14/789	1.78	0.83–3.84	0.14
Constipation	4/818	4/735	0.88	0.10–7.90	0.91
Cough	1/843	2/708	0.52	0.02–14.73	0.70
Diarrhea	63/1144	25/1007	1.95	1.10–3.47	0.02
Dyspnea	42/855	7/789	5.32	2.39–11.84	<0.01
Fatigue	71/1004	46/939	1.86	1.19–2.91	<0.01
Hypertension	75/560	32/427	2.94	0.62–13.96	0.18
Insomnia	20/843	12/708	1.30	0.62–2.71	0.48
Nausea	8/678	2/667	3.31	0.81–13.56	0.10
Peripheral edema	6/678	4/667	1.25	0.36–4.27	0.73
Pneumonia	173/1144	117/1007	1.31	1.06–1.63	0.01
Pyrexia	19/967	12/885	1.47	0.73–2.98	0.28
Upper respiratory tract infection	44/1144	22/1007	1.97	1.02–3.79	0.04

## Data Availability

Data sharing is not applicable to this article.

## Supplementary Materials

The following supporting information can be downloaded at https://www.mdpi.com/article/10.3390/jpm14040360/s1, Table S1: PRISMA 2020 Checklist; Table S2: PRISMA 2020 for Abstract Checklist; Table S3: Inclusion and exclusion criteria of included studies; Table S4: Search strategies; Table S5: Treatment regimens from the randomized controlled trials included in this systematic review and meta-analysis; Figure S1: Any grade of adverse events. (A) Anemia. (B) Arthralgia. (C) Asthenia. (D) Back pain. (E) Bronchitis. (F) Constipation. (G) Cough. (H) Diarrhea. (I) Dyspnea. (J). Fatigue. (K) Febrile neutropenia. (L) Hypertension. (M) Insomnia. (N) Lymphopenia. (O) Nausea. (P) Neutropenia. (Q) Peripheral edema. (R) Pneumonia. (S) Pyrexia. (T) Thrombocytopenia. (U) Upper respiratory tract infection; Figure S2: Grade ≥ 3 of adverse events. (A) Anemia. (B) Arthralgia. (C) Asthenia. (D) Back pain. (E) Bronchitis. (F) Constipation. (G) Cough. (H) Diarrhea. (I) Dyspnea. (J) Fatigue. (K) Febrile neutropenia. (L) Hypertension. (M) Insomnia. (N) Lymphopenia. (O) Nausea. (P) Neutropenia. (Q) Peripheral edema. (R) Pneumonia. (S) Pyrexia. (T) Thrombocytopenia. (U) Upper respiratory tract infection; Figure S3: Leave-one-out sensitivity analyses. (A1) Progression-free survival. (A2) AKT Mutation progression-free survival. (B1) Overall survival. (B2) AKT Mutation Overall survival. Any adverse effects. (C) Alopecia. (D) Diarrhea. (E) Fatigue. (F) Nausea. (G) Neuropathy. (H) Rash. (I) Stomatitis. (L) Vomiting; Figure S4: Leave-one-out sensitivity analyses. (A). Progression-free survival. (B). Overall survival; Figure S5: Heterogeneity analysis. A Funnel plot of Overall survival; Figure S6: Methodological quality summary using RoB2. (A) Risk of bias domains. (B) Overall risk of bias.

## References

[1] Morandi F., Horenstein A.L., Costa F., Giuliani N., Pistoia V., Malavasi F. (2018). CD38: A Target for Immunotherapeutic Approaches in Multiple Myeloma. Front. Immunol..

[2] Kuehl W.M., Bergsagel P.L. (2002). Multiple Myeloma: Evolving Genetic Events and Host Interactions. Nat. Rev. Cancer.

[3] Xu S., De Veirman K., De Becker A., Vanderkerken K., Van Riet I. (2018). Mesenchymal Stem Cells in Multiple Myeloma: A Therapeutical Tool or Target?. Leukemia.

[4] Salomon-Perzyński A., Jamroziak K., Głodkowska-Mrówka E. (2021). Clonal Evolution of Multiple Myeloma—Clinical and Diagnostic Implications. Diagnostics.

[5] Yavorkovsky L.L. (2021). Smoldering Multiple Myeloma 40 Years Later: A Story of Unintended Disease. Expert Rev. Hematol..

[6] Schmidt T., Callander N. (2020). Diagnosis and Management of Monoclonal Gammopathy and Smoldering Multiple Myeloma. J. Natl. Compr. Cancer Netw. JNCCN.

[7] International Myeloma Working Group (2003). Criteria for the Classification of Monoclonal Gammopathies, Multiple Myeloma and Related Disorders: A Report of the International Myeloma Working Group. Br. J. Haematol..

[8] Landgren O., Kyle R.A., Pfeiffer R.M., Katzmann J.A., Caporaso N.E., Hayes R.B., Dispenzieri A., Kumar S., Clark R.J., Baris D. (2009). Monoclonal Gammopathy of Undetermined Significance (MGUS) Consistently Precedes Multiple Myeloma: A Prospective Study. Blood.

[9] Zingone A., Kuehl W.M. (2011). Pathogenesis of Monoclonal Gammopathy of Undetermined Significance (MGUS) and Progression to Multiple Myeloma. Semin. Hematol..

[10] Kyle R.A., Durie B.G.M., Rajkumar S.V., Landgren O., Blade J., Merlini G., Kröger N., Einsele H., Vesole D.H., Dimopoulos M. (2010). Monoclonal Gammopathy of Undetermined Significance (MGUS) and Smoldering (Asymptomatic) Multiple Myeloma: IMWG Consensus Perspectives Risk Factors for Progression and Guidelines for Monitoring and Management. Leukemia.

[11] Rajkumar S.V., Dimopoulos M.A., Palumbo A., Blade J., Merlini G., Mateos M.-V., Kumar S., Hillengass J., Kastritis E., Richardson P. (2014). International Myeloma Working Group Updated Criteria for the Diagnosis of Multiple Myeloma. Lancet Oncol..

[12] Abramson H.N. (2023). Immunotherapy of Multiple Myeloma: Current Status as Prologue to the Future. Int. J. Mol. Sci..

[13] Bianchi G., Ghobrial I.M. (2012). Does My Patient with a Serum Monoclonal Spike Have Multiple Myeloma? Hematol. Oncol. Clin. N. Am..

[14] Phekoo K.J., Schey S.A., Richards M.A., Bevan D.H., Bell S., Gillett D., Møller H. (2004). Consultant Haematologists, South Thames Haematology Specialist Committee A Population Study to Define the Incidence and Survival of Multiple Myeloma in a National Health Service Region in UK. Br. J. Haematol..

[15] Palumbo A., Anderson K. (2011). Multiple Myeloma. N. Engl. J. Med..

[16] Rajkumar S.V. (2020). Multiple Myeloma: 2020 Update on Diagnosis, Risk-Stratification and Management. Am. J. Hematol..

[17] Palumbo A., Attal M., Roussel M. (2011). Shifts in the Therapeutic Paradigm for Patients Newly Diagnosed with Multiple Myeloma: Maintenance Therapy and Overall Survival. Clin. Cancer Res..

[18] Nakamura Z.M., Vohra S.N., Jensen C.E., Nyrop K.A., Deal A.M., Heiling H.M., Mangieri N.J., Grant S.J., Lichtman E.I., Rubinstein S.M. (2022). Prevalence and Clinical Correlates of Cognitive Impairment in Adults with Plasma Cell Disorders. J. Geriatr. Oncol..

[19] Zhou L., Yu Q., Wei G., Wang L., Huang Y., Hu K., Hu Y., Huang H. (2021). Measuring the Global, Regional, and National Burden of Multiple Myeloma from 1990 to 2019. BMC Cancer.

[20] Kumar S.K., Rajkumar S.V., Dispenzieri A., Lacy M.Q., Hayman S.R., Buadi F.K., Zeldenrust S.R., Dingli D., Russell S.J., Lust J.A. (2008). Improved Survival in Multiple Myeloma and the Impact of Novel Therapies. Blood.

[21] Kazandjian D. (2016). Multiple Myeloma Epidemiology and Survival: A Unique Malignancy. Semin. Oncol..

[22] Fonseca R., Abouzaid S., Bonafede M., Cai Q., Parikh K., Cosler L., Richardson P. (2017). Trends in Overall Survival and Costs of Multiple Myeloma, 2000–2014. Leukemia.

[23] Moreau P., Zamagni E., Mateos M.-V. (2019). Treatment of Patients with Multiple Myeloma Progressing on Frontline-Therapy with Lenalidomide. Blood Cancer J..

[24] Ho M., Bianchi G., Anderson K.C. (2020). Proteomics-Inspired Precision Medicine for Treating and Understanding Multiple Myeloma. Expert Rev. Precis. Med. Drug Dev..

[25] Brenner H., Gondos A., Pulte D. (2008). Recent Major Improvement in Long-Term Survival of Younger Patients with Multiple Myeloma. Blood.

[26] van de Donk N.W.C.J., Richardson P.G., Malavasi F. (2018). CD38 Antibodies in Multiple Myeloma: Back to the Future. Blood.

[27] Sarosiek S., Sanchorawala V. (2019). Treatment Options For Relapsed/Refractory Systemic Light-Chain (AL) Amyloidosis: Current Perspectives. J. Blood Med..

[28] Bhatt P., Kloock C., Comenzo R. (2023). Relapsed/Refractory Multiple Myeloma: A Review of Available Therapies and Clinical Scenarios Encountered in Myeloma Relapse. Curr. Oncol..

[29] Martin T.G., Corzo K., Chiron M., van de Velde H., Abbadessa G., Campana F., Solanki M., Meng R., Lee H., Wiederschain D. (2019). Therapeutic Opportunities with Pharmacological Inhibition of CD38 with Isatuximab. Cells.

[30] Chillemi A., Quarona V., Antonioli L., Ferrari D., Horenstein A.L., Malavasi F. (2017). Roles and Modalities of Ectonucleotidases in Remodeling the Multiple Myeloma Niche. Front. Immunol..

[31] Malavasi F., Deaglio S., Funaro A., Ferrero E., Horenstein A.L., Ortolan E., Vaisitti T., Aydin S. (2008). Evolution and Function of the ADP Ribosyl Cyclase/CD38 Gene Family in Physiology and Pathology. Physiol. Rev..

[32] Munshi C., Aarhus R., Graeff R., Walseth T.F., Levitt D., Lee H.C. (2000). Identification of the Enzymatic Active Site of CD38 by Site-Directed Mutagenesis. J. Biol. Chem..

[33] Lapietra G., Fazio F., Petrucci M.T. (2022). Race for the Cure: From the Oldest to the Newest Monoclonal Antibodies for Multiple Myeloma Treatment. Biomolecules.

[34] Page M.J., McKenzie J.E., Bossuyt P.M., Boutron I., Hoffmann T.C., Mulrow C.D., Shamseer L., Tetzlaff J.M., Akl E.A., Brennan S.E. (2021). The PRISMA 2020 Statement: An Updated Guideline for Reporting Systematic Reviews. BMJ.

[35] Ouzzani M., Hammady H., Fedorowicz Z., Elmagarmid A. (2016). Rayyan—A Web and Mobile App for Systematic Reviews. Syst. Rev..

[36] Common Terminology Criteria for Adverse Events (CTCAE) | Protocol Development | CTEP. https://ctep.cancer.gov/protocoldevelopment/electronic_applications/ctc.htm.

[37] Sterne J.A.C., Savović J., Page M.J., Elbers R.G., Blencowe N.S., Boutron I., Cates C.J., Cheng H.-Y., Corbett M.S., Eldridge S.M. (2019). RoB 2: A Revised Tool for Assessing Risk of Bias in Randomised Trials. BMJ.

[38] Higgins J.P.T., Thompson S.G., Deeks J.J., Altman D.G. (2003). Measuring Inconsistency in Meta-Analyses. BMJ.

[39] IntHout J., Ioannidis J.P.A., Borm G.F. (2014). The Hartung-Knapp-Sidik-Jonkman Method for Random Effects Meta-Analysis Is Straightforward and Considerably Outperforms the Standard DerSimonian-Laird Method. BMC Med. Res. Methodol..

[40] DerSimonian R., Laird N. (1986). Meta-Analysis in Clinical Trials. Control. Clin. Trials.

[41] Egger M., Davey Smith G., Schneider M., Minder C. (1997). Bias in Meta-Analysis Detected by a Simple, Graphical Test. BMJ.

[42] Dimopoulos M.A., Terpos E., Boccadoro M., Delimpasi S., Beksac M., Katodritou E., Moreau P., Baldini L., Symeonidis A., Bila J. (2021). Daratumumab plus Pomalidomide and Dexamethasone versus Pomalidomide and Dexamethasone Alone in Previously Treated Multiple Myeloma (APOLLO): An Open-Label, Randomised, Phase 3 Trial. Lancet Oncol..

[43] Richardson P.G., Perrot A., San-Miguel J., Beksac M., Spicka I., Leleu X., Schjesvold F., Moreau P., Dimopoulos M.A., Huang J.S.-Y. (2022). Isatuximab plus Pomalidomide and Low-Dose Dexamethasone versus Pomalidomide and Low-Dose Dexamethasone in Patients with Relapsed and Refractory Multiple Myeloma (ICARIA-MM): Follow-up Analysis of a Randomised, Phase 3 Study. Lancet Oncol..

[44] Martin T., Dimopoulos M.-A., Mikhael J., Yong K., Capra M., Facon T., Hajek R., Špička I., Baker R., Kim K. (2023). Isatuximab, Carfilzomib, and Dexamethasone in Patients with Relapsed Multiple Myeloma: Updated Results from IKEMA, a Randomized Phase 3 Study. Blood Cancer J..

[45] Lu J., Fu W., Li W., Hu J., An G., Wang Y., Fu C., Chen L., Jin J., Cen X. (2021). Daratumumab, Bortezomib, and Dexamethasone Versus Bortezomib and Dexamethasone in Chinese Patients with Relapsed or Refractory Multiple Myeloma: Phase 3 LEPUS (MMY3009) Study. Clin. Lymphoma Myeloma Leuk..

[46] Dimopoulos M.A., Oriol A., Nahi H., San-Miguel J., Bahlis N.J., Usmani S.Z., Rabin N., Orlowski R.Z., Suzuki K., Plesner T. (2023). Overall Survival With Daratumumab, Lenalidomide, and Dexamethasone in Previously Treated Multiple Myeloma (POLLUX): A Randomized, Open-Label, Phase III Trial. J. Clin. Oncol..

[47] Sonneveld P., Chanan-Khan A., Weisel K., Nooka A.K., Masszi T., Beksac M., Spicka I., Hungria V., Munder M., Mateos M.-V. (2023). Overall Survival With Daratumumab, Bortezomib, and Dexamethasone in Previously Treated Multiple Myeloma (CASTOR): A Randomized, Open-Label, Phase III Trial. J. Clin. Oncol..

[48] Overdijk M.B., Jansen J.H.M., Nederend M., Lammerts van Bueren J.J., Groen R.W.J., Parren P.W.H.I., Leusen J.H.W., Boross P. (2016). The Therapeutic CD38 Monoclonal Antibody Daratumumab Induces Programmed Cell Death via Fcγ Receptor-Mediated Cross-Linking. J. Immunol..

[49] Gozzetti A., Ciofini S., Simoncelli M., Santoni A., Pacelli P., Raspadori D., Bocchia M. (2022). Anti CD38 Monoclonal Antibodies for Multiple Myeloma Treatment. Hum. Vaccines Immunother..

[50] Melis J.P.M., Strumane K., Ruuls S.R., Beurskens F.J., Schuurman J., Parren P.W.H.I. (2015). Complement in Therapy and Disease: Regulating the Complement System with Antibody-Based Therapeutics. Mol. Immunol..

[51] Taylor R.P., Lindorfer M.A. (2016). Cytotoxic Mechanisms of Immunotherapy: Harnessing Complement in the Action of Anti-Tumor Monoclonal Antibodies. Semin. Immunol..

[52] Saltarella I., Desantis V., Melaccio A., Solimando A.G., Lamanuzzi A., Ria R., Storlazzi C.T., Mariggiò M.A., Vacca A., Frassanito M.A. (2020). Mechanisms of Resistance to Anti-CD38 Daratumumab in Multiple Myeloma. Cells.

[53] van de Donk N.W.C.J., Usmani S.Z. (2018). CD38 Antibodies in Multiple Myeloma: Mechanisms of Action and Modes of Resistance. Front. Immunol..

[54] Schmidt R.E., Gessner J.E. (2005). Fc Receptors and Their Interaction with Complement in Autoimmunity. Immunol. Lett..

[55] de Weers M., Tai Y.-T., van der Veer M.S., Bakker J.M., Vink T., Jacobs D.C.H., Oomen L.A., Peipp M., Valerius T., Slootstra J.W. (2011). Daratumumab, a Novel Therapeutic Human CD38 Monoclonal Antibody, Induces Killing of Multiple Myeloma and Other Hematological Tumors. J. Immunol..

[56] Tay M.Z., Wiehe K., Pollara J. (2019). Antibody-Dependent Cellular Phagocytosis in Antiviral Immune Responses. Front. Immunol..

[57] Lo Nigro C., Macagno M., Sangiolo D., Bertolaccini L., Aglietta M., Merlano M.C. (2019). NK-Mediated Antibody-Dependent Cell-Mediated Cytotoxicity in Solid Tumors: Biological Evidence and Clinical Perspectives. Ann. Transl. Med..

[58] Ramírez-Labrada A., Pesini C., Santiago L., Hidalgo S., Calvo-Pérez A., Oñate C., Andrés-Tovar A., Garzón-Tituaña M., Uranga-Murillo I., Arias M.A. (2022). All About (NK Cell-Mediated) Death in Two Acts and an Unexpected Encore: Initiation, Execution and Activation of Adaptive Immunity. Front. Immunol..

[59] Sordo-Bahamonde C., Lorenzo-Herrero S., Payer Á.R., Gonzalez S., López-Soto A. (2020). Mechanisms of Apoptosis Resistance to NK Cell-Mediated Cytotoxicity in Cancer. Int. J. Mol. Sci..

[60] Brandsma A.M., Bondza S., Evers M., Rösner T., Valerius T., ten Broeke T. (2019). Potent Fc Receptor Signaling by IgA Leads to Superior Killing of Cancer Cells by Neutrophils Compared to IgG. Front. Immunol..

[61] Zent C.S., Elliott M.R. (2017). Maxed out Macs: Physiologic Cell Clearance as a Function of Macrophage Phagocytic Capacity. FEBS J..

[62] Kamen L., Myneni S., Langsdorf C., Kho E., Ordonia B., Thakurta T., Zheng K., Song A., Chung S. (2019). A Novel Method for Determining Antibody-Dependent Cellular Phagocytosis. J. Immunol. Methods.

[63] Zhu C., Song Z., Wang A., Srinivasan S., Yang G., Greco R., Theilhaber J., Shehu E., Wu L., Yang Z.-Y. (2020). Isatuximab Acts Through Fc-Dependent, Independent, and Direct Pathways to Kill Multiple Myeloma Cells. Front. Immunol..

[64] Shen F., Shen W. (2022). Isatuximab in the Treatment of Multiple Myeloma: A Review and Comparison with Daratumumab. Technol. Cancer Res. Treat..

[65] Romano A., Storti P., Marchica V., Di Raimondo F., Giuliani N. (2021). Mechanisms of Action of the New Antibodies in Use in Multiple Myeloma. Front. Oncol..

[66] Munshi N.C., Anderson L.D., Shah N., Madduri D., Berdeja J., Lonial S., Raje N., Lin Y., Siegel D., Oriol A. (2021). Idecabtagene Vicleucel in Relapsed and Refractory Multiple Myeloma. N. Engl. J. Med..

[67] Seitzler S., Finley-Oliver E., Simonelli C., Baz R. (2019). Quality of Life in Multiple Myeloma: Considerations and Recommendations. Expert Rev. Hematol..

[68] Fragola M. (2020). Patient-Reported Outcomes and Assessment of Quality of Life: A Focus on Multiple Myeloma. J. Adv. Pract. Oncol..

