# Supplementary material for: Efficacy and Safety of Anti-CD38 Monoclonal Antibodies in Patients with Relapsed or Refractory Multiple Myeloma: A Meta-Analysis of Randomized Clinical Trials
Source: J Pers Med. 2024 Mar 29;14(4):360. doi: 10.3390/jpm14040360 (PMC11051236; doi:10.3390/jpm14040360)

Supplementary Material

Supplementary Table S1 PRISMA 2020 Checklist.

Supplementary Table S2 PRISMA 2020 for Abstract Checklist.

Supplementary Table S3 Inclusion and exclusion criteria of included studies

Supplementary Table S4 Search strategies.

Supplementary Table S5 Treatment regimens from the randomized controlled trials included in this systematic review and meta-analysis

Supplementary Figure S1 Any grade of adverse events. **A.** Anemia. **B** Arthralgia. **C** Asthenia. **D** Back pain**. E.** Bronchitis. **F** Constipation**. G** Cough**. H** Diarrhea**. I** Dyspnea. **J**. Fatigue. **K.** Febrile neutropenia. **L**. Hypertension. **M**.Insomnia. **N**.Lymphopenia. **O**.Nausea. **P.**Neutropenia**. Q**.Peripheral edema. **R**.Pneumonia. **S.** Pyrexia **T.** Thrombocytopenia. **U**. Upper respiratory tract infection

Supplementary Figure S2 Grade ≧3 of adverse events. **A.** Anemia. **B** Arthralgia. **C** Asthenia. **D** Back pain**. E.** Bronchitis. **F** Constipation**. G** Cough**. H** Diarrhea**. I** Dyspnea. **J**. Fatigue. **K.** Febrile neutropenia. **L**. Hypertension. **M**.Insomnia. **N**.Lymphopenia. **O**.Nausea. **P.**Neutropenia**. Q**.Peripheral edema. **R**.Pneumonia. **S.** Pyrexia **T.** Thrombocytopenia. **U**. Upper respiratory tract infection

Supplementary Figure S3 Leave-one-out sensitivity analyses plots. **A.** Progression-free survival. **B.** Overall survival**.**

Supplementary Figure S4 Funnel plot of the overall survival outcome.

Supplementary Figure S5 Funnel plot of the progression-free survival outcome.

Supplementary Figure S6 Overall risk of bias

25 **Supplementary Table S1. PRISMA 2020 Checklist [34].**

Section and Topic	Item #	Checklist item	Location where item is reported
TITLE			
Title	1	Identify the report as a systematic review.	Page 1
ABSTRACT			
Abstract	2	See the PRISMA 2020 for Abstracts checklist.	Supplementary Materials: Table S3.
INTRODUCTION			
Rationale	3	Describe the rationale for the review in the context of existing knowledge.	Page 1
Objectives	4	Provide an explicit statement of the objective(s) or question(s) the review addresses.	Page 2
METHODS			
Eligibility criteria	5	Specify the inclusion and exclusion criteria for the review and how studies were grouped for the syntheses.	Pages 2-3
Information sources	6	Specify all databases, registers, websites, organisations, reference lists and other sources searched or consulted to identify studies. Specify the date when each source was last searched or consulted.	Page 3
Search strategy	7	Present the full search strategies for all databases, registers and websites, including any filters and limits used.	Supplementary Materials: Table S4 Search Strategies
Selection process	8	Specify the methods used to decide whether a study met the inclusion criteria of the review, including how many reviewers screened each record and each report retrieved, whether they worked independently, and if applicable, details of automation tools used in the process.	Page 3
Data collection process	9	Specify the methods used to collect data from reports, including how many reviewers collected data from each report, whether they worked independently, any processes for obtaining or confirming data from study investigators, and if applicable, details of automation tools used in the process.	Page 3

Data items	10a	List and define all outcomes for which data were sought. Specify whether all results that were compatible with each outcome domain in each study were sought (e.g. for all measures, time points, analyses), and if not, the methods used to decide which results to collect.	Pages 3
	10b	List and define all other variables for which data were sought (e.g. participant and intervention characteristics, funding sources). Describe any assumptions made about any missing or unclear information.	Page NO
Study risk of bias assessment	11	Specify the methods used to assess risk of bias in the included studies, including details of the tool(s) used, how many reviewers assessed each study and whether they worked independently, and if applicable, details of automation tools used in the process.	Page 4
Effect measures	12	Specify for each outcome the effect measure(s) (e.g. risk ratio, mean difference) used in the synthesis or presentation of results.	Page 4
Synthesis methods	13a	Describe the processes used to decide which studies were eligible for each synthesis (e.g. tabulating the study intervention characteristics and comparing against the planned groups for each synthesis (item #5)).	Pages 2 and 3
	13b	Describe any methods required to prepare the data for presentation or synthesis, such as handling of missing summary statistics, or data conversions.	Pages 4
	13c	Describe any methods used to tabulate or visually display results of individual studies and syntheses.	Page 3
	13d	Describe any methods used to synthesize results and provide a rationale for the choice(s). If meta-analysis was performed, describe the model(s), method(s) to identify the presence and extent of statistical heterogeneity, and software package(s) used.	Page 4
	13e	Describe any methods used to explore possible causes of heterogeneity among study results (e.g. subgroup analysis, meta-regression).	Pages 4
	13f	Describe any sensitivity analyses conducted to assess robustness of the synthesized results.	Pages 4
Reporting bias assessment	14	Describe any methods used to assess risk of bias due to missing results in a synthesis (arising from reporting biases).	Pages 4
Certainty assessment	15	Describe any methods used to assess certainty (or confidence) in the body of evidence for an outcome.	Pages 4
RESULTS			
Study selection	16a	Describe the results of the search and selection process, from the number of records identified in the search to the number of studies included in the review, ideally using a flow diagram.	Page 4-5<br>Figure 1

	16b	Cite studies that might appear to meet the inclusion criteria, but which were excluded, and explain why they were excluded.	Page NO
Study characteristics	17	Cite each included study and present its characteristics.	Pages 5-6<br>Table 1
Risk of bias in studies	18	Present assessments of risk of bias for each included study.	Supplementary Materials: Figure 4.
Results of individual studies	19	For all outcomes, present, for each study: (a) summary statistics for each group (where appropriate) and (b) an effect estimate and its precision (e.g. confidence/credible interval), ideally using structured tables or plots.	Pages 7-9<br>Figure 2 and 3
Results of syntheses	20a	For each synthesis, briefly summarise the characteristics and risk of bias among contributing studies.	Pages 7-9<br>Supplementary Materials: Figure 4
	20b	Present results of all statistical syntheses conducted. If meta-analysis was done, present for each the summary estimate and its precision (e.g. confidence/credible interval) and measures of statistical heterogeneity. If comparing groups, describe the direction of the effect.	Pages 7-9<br>Figure 2 and 3<br>Supplementary Materials: Figure 1, 2 and 3
	20c	Present results of all investigations of possible causes of heterogeneity among study results.	Page 9<br>Figure 4
	20d	Present results of all sensitivity analyses conducted to assess the robustness of the synthesized results.	Page 8-9<br>Supplementary Materials: Figure 2
Reporting biases	21	Present assessments of risk of bias due to missing results (arising from reporting biases) for each synthesis assessed.	Page NO
Certainty of evidence	22	Present assessments of certainty (or confidence) in the body of evidence for each outcome assessed.	Page 9
DISCUSSION			
Discussion	23a	Provide a general interpretation of the results in the context of other evidence.	Pages 9-11
	23b	Discuss any limitations of the evidence included in the review.	Page 10-11
	23c	Discuss any limitations of the review processes used.	Page 10-11
	23d	Discuss implications of the results for practice, policy, and future research.	Page 10
OTHER INFORMATION			

Registration and protocol	24a	Provide registration information for the review, including register name and registration number, or state that the review was not registered.	Page 2
	24b	Indicate where the review protocol can be accessed, or state that a protocol was not prepared.	Page 2
	24c	Describe and explain any amendments to information provided at registration or in the protocol.	Page 2
Support	25	Describe sources of financial or non-financial support for the review, and the role of the funders or sponsors in the review.	Page 12
Competing interests	26	Declare any competing interests of review authors.	Page 12
Availability of data, code and other materials	27	Report which of the following are publicly available and where they can be found: template data collection forms; data extracted from included studies; data used for all analyses; analytic code; any other materials used in the review.	Page 12

Supplementary Table S2. PRISMA 2020 for Abstract Checklist [34].

Section and Topic	Item #	Checklist item	Reported (Yes/No)
TITLE			
Title	1	Identify the report as a systematic review.	Yes
BACKGROUND			
Objectives	2	Provide an explicit statement of the main objective(s) or question(s) the review addresses.	Yes
METHODS			
Eligibility criteria	3	Specify the inclusion and exclusion criteria for the review.	No
Information sources	4	Specify the information sources (e.g. databases, registers) used to identify studies and the date when each was last searched.	Yes
Risk of bias	5	Specify the methods used to assess risk of bias in the included studies.	No
Synthesis of results	6	Specify the methods used to present and synthesise results.	No
RESULTS			
Included studies	7	Give the total number of included studies and participants and summarise relevant characteristics of studies.	Yes
Synthesis of results	8	Present results for main outcomes, preferably indicating the number of included studies and participants for each. If meta-analysis was done, report the summary estimate and confidence/credible	Yes

		interval. If comparing groups, indicate the direction of the effect (i.e. which group is favoured).	
DISCUSSION			
Limitations of evidence	9	Provide a brief summary of the limitations of the evidence included in the review (e.g. study risk of bias, inconsistency and imprecision).	No
Interpretation	10	Provide a general interpretation of the results and important implications.	Yes
OTHER			
Funding	11	Specify the primary source of funding for the review.	No
Registration	12	Provide the register name and registration number.	No

Supplementary Table S3 Inclusion and exclusion criteria of included studies [42–47]

**Study**	**Inclusion Criteria**	**Exclusion Criteria**
APOLLO, 2021	1. Patients were aged 18 years or older, had relapsed or refractory multiple myeloma with measurable disease<br>2. An Eastern Cooperative Oncology Group performance status of 0–2<br>3. Received at least one previous line of therapy with both lenalidomide and a proteasome inhibitor<br>4. Partial response or better to one or more previous lines of antimyeloma therapy, and were refractory to lenalidomide if they had received only one previous line of treatment<br>5. Absolute neutrophil count of 1·0×10⁹ or more per L<br>6. Haemoglobin concentration of 7·5 g or more per dL,<br>7. Platelet count of 75×10⁹ or more per L if less than 50% of bone	1. Patients were aged 17 years or less<br>2. Previous therapy with any anti-CD38 monoclonal antibody.<br>3. Previous exposure to pomalidomide.<br>4. Subject has received antimyeloma treatment within 2 weeks or 5 pharmacokinetic half-lives of the treatment, whichever is longer, before the date of randomization. The only exception is emergency use of a short course of corticosteroids (equivalent of dexamethasone 40 mg/day for a maximum of 4 days) for palliative treatment before Cycle 1, Day 1 (C1D1).

	marrow nucleated cells were plasma cells (otherwise, a platelet count of $\geq 50\times10^9$ per L),<br>8. Alanine aminotransferase and aspartate aminotransferase concentrations of no more than 2·5 times the upper limit of normal,<br>9. Total bilirubin concentration of no more than 1·5 times the upper limit of normal,<br>10. Creatinine clearance of 30 mL or more per min,<br>11. Serum calcium concentration corrected for albumin of 14·0 mg or less per dL, or free ionised calcium concentration of 6·5 mg or less per dL	5. Previous allogenic stem cell transplant; or autologous stem cell transplantation (ASCT) within 12 weeks before C1D1.<br>6. History of malignancy (other than MM) within 3 years before the date of randomization (exceptions are squamous and basal cell carcinomas of the skin, carcinoma in situ of the cervix or breast, or other non-invasive lesion that in the opinion of the investigator, with concurrence with the sponsor's medical monitor, is considered cured with minimal risk of recurrence within 3 years).<br>7. Clinical signs of meningeal involvement of MM.<br>8. Chronic obstructive pulmonary disease (COPD) with a Forced Expiratory Volume in 1 second (FEV1) <50% of predicted normal. Note that FEV1 testing is required for subjects suspected of having COPD and subjects must be excluded if FEV1 <50% of predicted normal.<br>9. Clinically significant cardiac disease, including:<br>a. Myocardial infarction within 6 months, before C1D1 or unstable or uncontrolled condition (eg, unstable angina, congestive heart failure, New York Heart Association Class III-IV).<br>b. Cardiac arrhythmia (Common Terminology Criteria for Adverse Events [CTCAE] Grade 3 or higher) or clinically significant electrocardiogram (ECG) abnormalities.<br>c. Electrocardiogram showing a baseline QT interval as corrected QTc >470 msec.<br>10. Known active hepatitis A, B, or C.<br>11. Known HIV infection.<br>12. Gastrointestinal disease that may significantly alter the absorption of pomalidomide.<br>13. Subject has plasma cell leukemia (>2.0 × 109/L circulating plasma cells by standard differential) or Waldenström's macroglobulinemia or POEMS

		syndrome (polyneuropathy, organomegaly, endocrinopathy, monoclonal protein, and skin changes) or amyloidosis.<br>14. Any concurrent medical or psychiatric condition or disease (eg, active systemic infection, uncontrolled diabetes, acute diffuse infiltrative pulmonary disease) that is likely to interfere with the study procedures or results or that, in the opinion of the investigator, would constitute a hazard for participating in this study.<br>15. Ongoing ≥ Grade 2 peripheral neuropathy.<br>16. Subject had ≥Grade 3 rash during prior therapy.<br>17. Subject has had major surgery within 2 weeks before randomization, or has not fully recovered from an earlier surgery, or has surgery planned during the time the subject is expected to participate in the study or within 2 weeks after the last dose of study drug administration. Note: subjects with planned surgical procedures to be conducted under local anesthesia may participate. Kyphoplasty or vertebroplasty are not considered major surgery.<br>18. Pregnant or nursing women.
CASTOR, 2023	1. Must have had documented multiple myeloma<br>2. Subjects who are 18 years of age<br>3. Must have received at least 1 prior line of therapy for multiple myeloma<br>4. Must have had documented evidence of progressive disease as defined based on Investigator's determination of response of International Myeloma Working Group (IMWG) criteria on or after their last regimen<br>5. Must have an Eastern Cooperative Oncology Group Performance Status score of 0, 1, or 2<br>6. Must have achieved a response (partial response [PR] or better	1. Has received daratumumab or other anti-CD38 therapies previously<br>2. Is refractory to VELCADE or another PI, like ixazomib and carfilzomib (had progression of disease while receiving VELCADE therapy or within 60 days of ending VELCADE therapy or another PI therapy, like ixazomib and carfilzomib<br>3. Is intolerant to VELCADE (ie, discontinued due to any adverse event while on VELCADE treatment)<br>4. Has received anti-myeloma treatment within 2 weeks or 5 pharmacokinetic half-lives of the treatment, whichever is longer, before the date of randomization. The only exception is emergency use of a short course of corticosteroids

	based on investigator's determination of response by the IMWG criteria) to at least 1 prior regimen in the past	(equivalent of dexamethasone 40 milligram per day [mg/day] for a maximum of 4 days) before treatment. A list of anti-myeloma treatments with the corresponding pharmacokinetic half-lives is provided in the Site Investigational Product Procedures Manual (IPPM).<br>5. Has a history of malignancy (other than multiple myeloma) within 3 years before the date of randomization<br>6. Has any concurrent medical condition or disease (eg, active systemic infection) that is likely to interfere with study procedures
ICARIA-MM, 2022	1. Age superior or equal to 18 years or country's legal age of majority if the legal age was superior to 18 years old.<br>2. Participants had a documented diagnosis of multiple myeloma with evidence of measurable disease i.e. serum M protein superior or equal to 0.5 grams per decilitre (g/dL) measured using serum protein immunoelectrophoresis and or urine M protein superior or equal to 200 mg per 24 hours measured using urine protein immunoelectrophoresis.<br>3. Participants had received at least 2 prior lines of anti-myeloma therapy, which must include at least 2 consecutive cycles of lenalidomide and a proteasome inhibitor (bortezomib, carfilzomib or ixazomib) given alone or in combination.<br>4. Participants had failed treatment with lenalidomide and a proteasome inhibitor (bortezomib, carfilzomib, or ixazomib) alone or in combination (Intolerant, progression within 6 months after reaching Partial Response or better).<br>5. Participants had progressed on or within 60 days after end of previous therapy before to study entry, i.e., refractory to the last line of treatment.	1. Primary refractory multiple myeloma defined as participants who had never achieved at least a minimal response (MR) with any treatment during the disease course.<br>2. Free Light Chain measurable disease only.<br>3. Prior therapy with pomalidomide.<br>4. Any anti-myeloma drug treatment within 14 days before randomization, including dexamethasone.<br>5. Eastern Cooperative Oncology Group performance status superior to 2.<br>6. Platelets inferior to 75 000 cells per microliter (mcL) if inferior to 50% of bone marrow (BM) nucleated cells are plasma cells, and inferior to 30 000 cells per mcL if superior or equal to 50% of BM nucleated cells are plasma cells. Platelet transfusion was not allowed within three days before the screening visit.<br>7. Absolute neutrophil count inferior to 1000 per mcL (1*10^9/L).<br>8. Creatinine clearance inferior to 30 mL per minute (Modification of Diet in Renal Disease [MDRD] Formula).<br>9. Total bilirubin superior to 2*ULN (Upper Limit of Normal).<br>10. Corrected serum calcium superior to 14 milligrams per deciliter (mg/dL) (superior to 3.5 millimoles per liter (mmol/L).<br>11. Aspartate aminotransferase (AST) and/or Alanine Aminotransferase (ALT) superior to 3*ULN.

		12. Hypersensitivity to immunomodulatory drugs (IMiDs) (thalidomide or lenalidomide) defined as any hypersensitivity reaction leading to stop IMiDs within the 2 first cycles or toxicity, which does meet intolerance definition.<br>13. Hypersensitivity to dexamethasone, sucrose histidine (as base and hydrochloride salt), and polysorbate 80 or any of the components of study therapy that are not amenable to premedication with steroids, or H2 blockers that would prohibit further treatment with these agents.<br>14. Significant cardiac dysfunction; myocardial infarction within 12 months; unstable, poorly controlled angina pectoris.<br>15. Pregnant or breastfeeding woman or female who intends to become pregnant during the participation in the study.<br>16. Male participants who disagreed to practice true abstinence or disagreed to use a condom during sexual contact with a pregnant female or a female of childbearing potential while participating in the study, during dose interruptions and at least 3 or 5 months following study treatment discontinuation, even if he had undergone a successful vasectomy.<br>17. All participants who disagreed to refrain from donating blood while on study treatment and for 4 weeks after discontinuation from this study treatment.
IKEMA, 2023	1. Participants with MM previously treated with prior 1 to 3 lines and with measurable serum M-protein (>= 0.5 gram/deciliter) and/or urine M-protein (>= 200 milligram/24 hours).	1. Participants previously pretreated with carfilzomib, who never achieved at least one minor response during previous therapies and/or last previous therapy completed within 14 last days.<br>2. Participants with serum free light chain (FLC) measurable disease only.<br>3. Participants less than 18 years old, participants with Eastern Cooperative Oncology Group performance status more than 2.<br>4. Participants with inadequate biological tests.<br>5. Participants with myocardial infarction, severe/unstable angina pectoris, coronary/peripheral artery bypass graft, New York Heart Association class III or IV congestive heart failure, superior or equal to grade 3 arrhythmias, stroke or transient ischemic attack within last 6 months, and/or left ventricular ejection fraction lower than 40%.

		6. Participants with previous cancer unless disease free for more than 5 years or in situ cancer curatively treated.<br>7. Participants with known acquired immunodeficiency syndrome related illness or human immunodeficiency virus requiring antiretroviral treatment, or hepatitis A, B, or C active infection.<br>8. Women of childbearing potential or male participant with women of childbearing potential who do not agree to use highly effective method of birth control.
LEPUS, 2021	1. Documented multiple myeloma (MM) as defined by the criteria: monoclonal plasma cells in the bone marrow greater than or equal to (>=) 10 percent (%) at some point in the participant's disease course or presence of a biopsy-proven plasmacytoma<br>2. Eligible patients were ≥18 years of age<br>3. Received at least 1 prior line of therapy for MM<br>4. Documented evidence of progressive disease (PD) based on investigator's determination of response as defined by the International Myeloma Working Group (IMWG) criteria on or after their last regimen<br>5. Achieved a response (partial response [PR] or better based on investigator's determination of response by the IMWG criteria) to at least 1 prior regimen<br>6. Eastern Cooperative Oncology Group (ECOG) Performance Status score of 0, 1, or 2	1. Received daratumumab or other anti-CD38 therapies<br>2. Refractory to Velcade, or another proteasome inhibitor (PI), like ixazomib and carfilzomib (ie, participant had progression of disease while receiving Velcade therapy or within 60 days of ending Velcade therapy, or another PI, like ixazomib and carfilzomib, etc)<br>3. Intolerant to Velcade (that is [ie], discontinued due to any adverse event while on Velcade treatment)<br>4. Planning to undergo a stem cell transplant prior to progression of disease on this study, that is ie, these participants should not be enrolled in order to reduce disease burden prior to transplant<br>5. History of malignancy (other than MM) within 3 years before the date of randomization
POLLUX, 2023	1. 18 Years and older<br>2. Must have documented multiple myeloma and measurable disease<br>3. Must have received at least 1 prior line of therapy for multiple myeloma and achieved a response (partial response or better) to at least one prior regimen<br>4. Must have documented evidence of progressive disease as defined by the International Myeloma Working Group criteria on or after	1. Has received any of the following therapies: daratumumab or other anti-CD38 therapies<br>2. Has received anti-myeloma treatment within 2 weeks or 5 pharmacokinetic half-lives of the treatment<br>3. Disease shows evidence of refractoriness or intolerance to lenalidomide or if previously treated with a lenalidomide-containing regimen the participant is excluded if he or she

	their last regimen<br>5. Must have an Eastern Cooperative Oncology Group Performance Status score of 0, 1, or 2<br>6. If a participant has received subsequent anticancer therapy (salvage therapy), the participant must have a "wash-out period" defined as 2 weeks or 5 pharmacokinetic half-lives of the treatment, whichever is longer, before the planned start date of daratumumab monotherapy. The only exception is the emergency use of a short course of corticosteroids (equivalent of dexamethasone 40 milligram per day for a maximum of 4 days) before Daratumumab monotherapy	discontinued due to any adverse event related to prior lenalidomide treatment<br>4. Has received autologous stem cell transplantation within 12 weeks before the date of randomization, or previously received an allogenic stem cell transplant (regardless of timing), or planning to undergo a stem cell transplant prior to progression of disease<br>5. History of malignancy (other than multiple myeloma) within 5 years before the first dose of daratumumab monotherapy (exceptions are squamous and basal cell carcinomas of the skin and carcinoma in situ of the cervix, or breast, or other non-invasive lesion, that in the opinion of the investigator, with concurrence with the sponsor's medical monitor, is considered cured with minimal risk of recurrence within 5 years)

Supplementary Table S4 Search Strategies

**Database**	**Search Strategy**
**PubMed**	("Multiple Myeloma"[MeSH] OR "Myeloma, Multiple"[MeSH] OR "Myeloma"[MeSH] AND "Multiple") AND (Daratumumab[MeSH] OR Isatuximab[MeSH] OR "CD38 inhibitors"[MeSH] OR CD38[MeSH]) AND (clinical trial[Publication Type] OR randomized controlled trial[Publication Type])
**Embase**	((Multiple Myeloma OR "Multiple Myeloma" OR "Myeloma-Multiple") ab title OR (Multiple Myeloma OR "Multiple Myeloma" OR "Myeloma-Multiple") keyword) AND ((Daratumumab OR Isatuximab OR "CD38 inhibitors" OR CD38) ab title OR (Daratumumab OR Isatuximab OR "CD38 inhibitors" OR CD38) keyword) AND (clinical trial[pt] OR "controlled clinical trial"[pt] OR randomized controlled trial[pt] OR (random OR randomly OR randomized OR randomized OR randomization)[pt])
**The Cochrane Library**	(Multiple Myeloma OR "Multiple Myeloma" OR "Myeloma-Multiple") AND ((Daratumumab OR Isatuximab OR CD38 inhibitors OR CD38) AND (RCT OR random OR randomly OR randomized OR randomized OR randomization OR Randomized Controlled Trial OR Randomized Clinical Trial))

Supplementary Table S5 Treatment regimens from the randomized controlled trials included in this systematic review and meta-analysis [42–47].

Study	Treatment regimes
APOLLO, 2021	Daratumumab at a dose of 16 mg/kg administered as an IV infusion (Dara IV) or 1800 mg subcutaneously (Dara SC) at weekly intervals (QW) for 8 weeks, then every 2 weeks (Q2W) for an additional 16 weeks, then every 4 weeks (Q4W) thereafter. Pomalidomide 4 mg orally (PO) on Days 1 through 21 of each 28-day cycle Dexamethasone 40 mg (20 mg for patients ≥75 years of age) orally, once daily on Days 1, 8, 15, and 22 of each 28-day treatment cycle
CASTOR, 2023	Drug: Daratumumab<br>● Daratumumab be administered as an IV infusion or 16 mg/kg weekly for the first 3 cycles, on Day 1 of Cycles 4-9, and then every 4 weeks thereafter. As per protocol amendment-6 participants receiving treatment with daratumumab IV will have the option to switch to daratumumab SC 1800 mg on Day 1 of any cycle, at the discretion of the investigator.<br>Drug: VELCADE (Bortezomib)<br>● VELCADE be administered at a dose of 1.3 mg/m2 subcutaneously (SC) on Days 1, 4, 8 and 11 of each 21-day cycle. Eight VELCADE treatment cycles are to be administered.<br>● Other Names:<br>○ VELCADE<br>Drug: Dexamethasone<br>● Dexamethasone be administered orally at 20 mg on Days 1, 2, 4, 5, 8, 9, 11 and 12 of the first 8 VELCADE treatment cycles.
ICARIA-MM, 2022	Participants received isatuximab 10 milligrams per kilogram (mg/kg) intravenous (IV) infusion on Days 1, 8, 15, and 22 at Cycle 1, and then on Days 1 and 15 of subsequent cycles plus pomalidomide 4 mg PO on Days 1 to 21 of each 28-day treatment cycle and dexamethasone 40 mg (participants >= 75 years of age received 20 mg dexamethasone), PO or IV on Day 1, 8, 15, 22 of each 28-day treatment cycle until disease progression or unacceptable toxicity or participant's wish to discontinue study treatment, or any other reason, whichever comes first
IKEMA, 2023	Isatuximab (intravenous) on day 1, 8, 15 and 22 of 1st cycle, then on day 1 and 15 of subsequent cycles in combination with carfilzomib (intravenous) on day 1, 2, 8, 9, 15 and 16 + dexamethasone (intravenous or by mouth [po]) on day 1, 2, 8, 9, 15, 16, 22 and 23 of a 28 day cycle.
LEPUS, 2021	Participants received daratumumab weekly for the first 3 cycles, every 3 weeks (q3w) on Day 1 of Cycles 4-9 as an intravenous (IV) infusion at a dose of 16 milligram per kilogram (mg/kg) or will have the option to switch to daratumumab subcutaneously (SC) on Day 1 of any cycle, and then every 4 weeks (q4w) thereafter, Velcade at a dose of 1.3 milligram per square meter (mg/m^2) subcutaneous (SC) on Days 1, 4, 8 and 11 of each 21-day

	cycle (up to 8 treatment cycles) and dexamethasone (Dex) orally (PO) at 20 milligram (mg) on Days 1, 2, 4, 5, 8, 9, 11 and 12 of the 8 Velcade treatment cycles.
POLLUX, 2023	Drug: Daratumumab<br>● Daratumumab 16mg/kg be administered as an intravenous (IV) infusion (into the vein) as per the following schedule: once a week during treatment cycles 1 and 2; every 2 weeks during treatment cycles 3 to 6; and every 4 weeks for cycles 7 and onwards. Following amendment 8, participants receiving daratumumab IV have the option to switch to daratumumab subcutaneous (SC) 1800 mg/dose until documented progression, unacceptable toxicity, or the end of study on Day 1 of any cycle, at the discretion of the investigator.<br>Drug: Lenalidomide<br>● Lenalidomide be administered at a dose of 25 mg orally (by mouth) on Days 1 through 21 of each treatment cycle.<br>Drug: Dexamethasone<br>● Dexamethasone (or equivalent in accordance with local standards) be administered as a total dose of 40 mg weekly (or 20 mg weekly for participants > 75 years old or with a body mass index < 18.5).

Supplementary Figure 1 Any grade of adverse events. **A.** Anemia. **B** Arthralgia. **C** Asthenia. **D** Back pain**. E.** Bronchitis. **F** Constipation**. G** Cough**. H** Diarrhea**. I** Dyspnea. **J**. Fatigue. **K.** Febrile neutropenia. **L**. Hypertension.

M.Insomnia. **N**.Lymphopenia. **O**.Nausea. **P**.Neutropenia**.** **Q**.Peripheral edema. **R**.Pneumonia. **S.** Pyrexia **T.** Thrombocytopenia. **U**. Upper respiratory tract infection.

A Anemia [42–47].

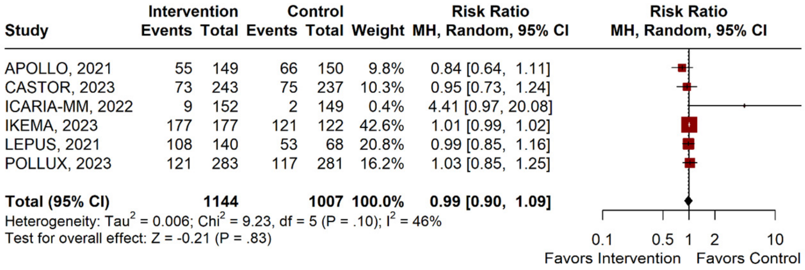

B Arthralgia [43–44,46–47].

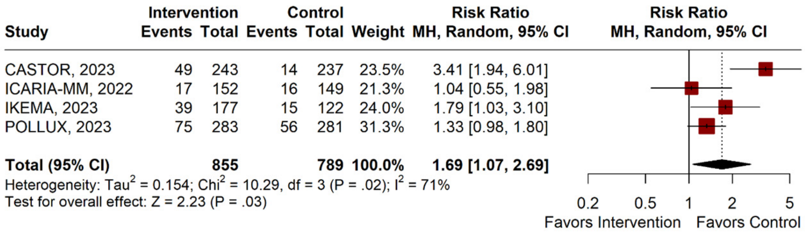

C Asthenia [42–44,46–47].

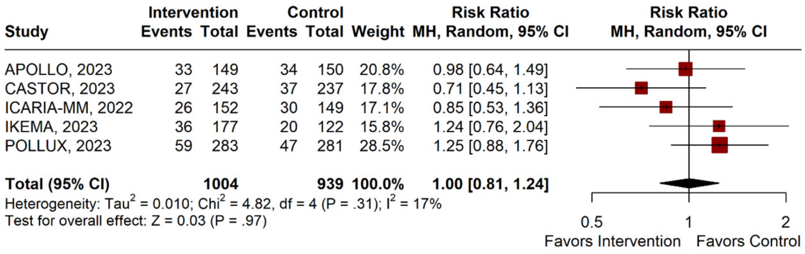

D Back pain [43–44, 46–47].

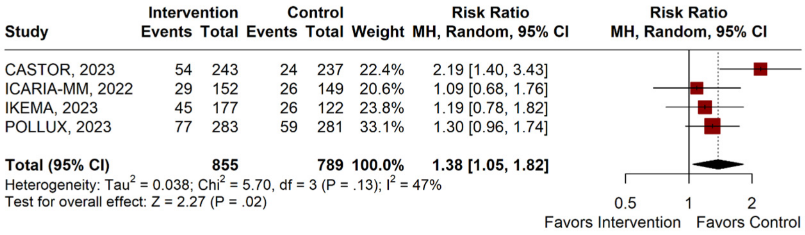

E. Bronchitis [43–44, 46–47].

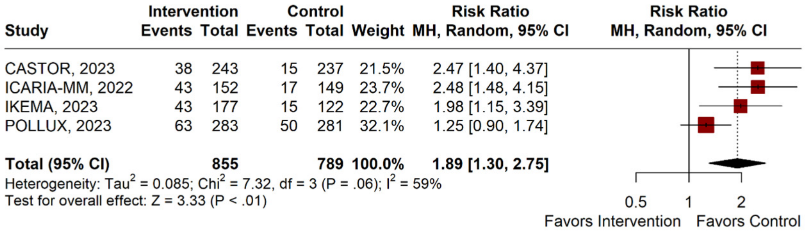

F Constipation [43,45–47].

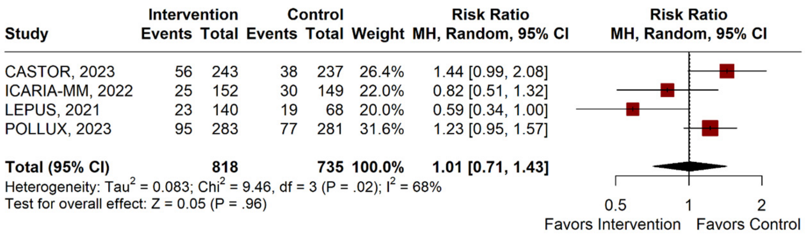

G Cough [44–47]..

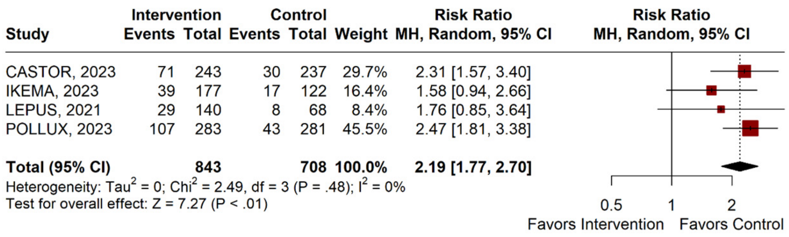

H Diarrhea [42–47].

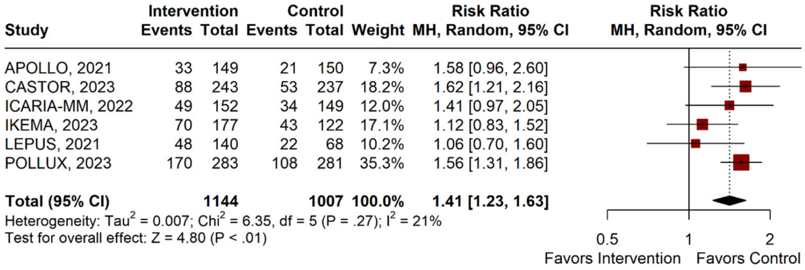

I Dyspnea [43–44, 46–47].

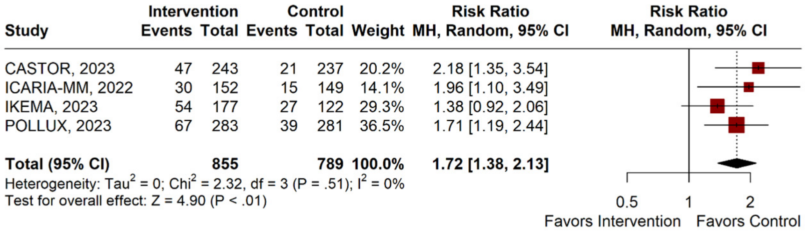

J. Fatigue [42–44, 46–47].

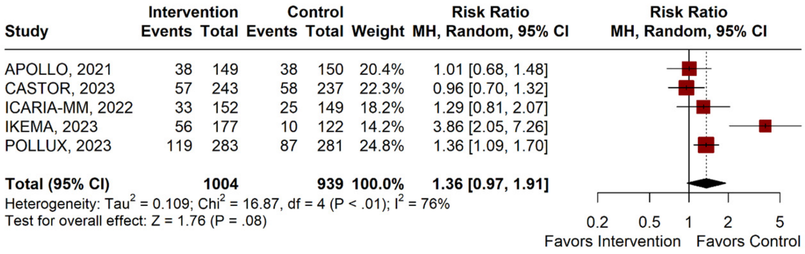

K. Febrile neutropenia [42,43,46].

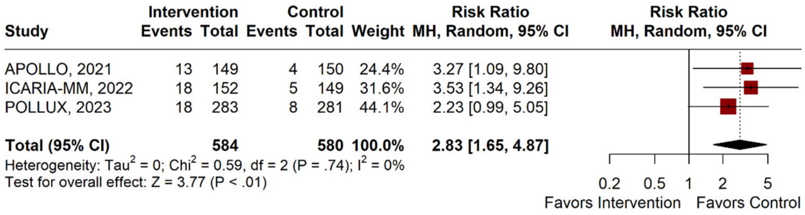

L. Hypertension [44,45,47].

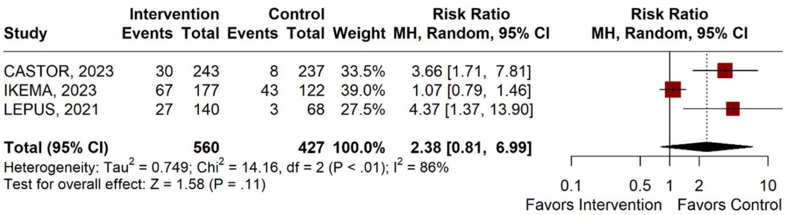

M. Insomnia [44–47].

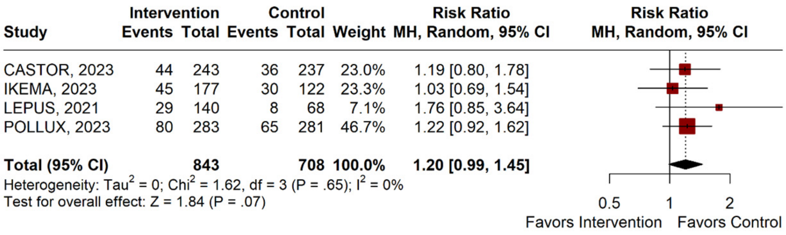

N. Lymphopenia [42,45–47]..

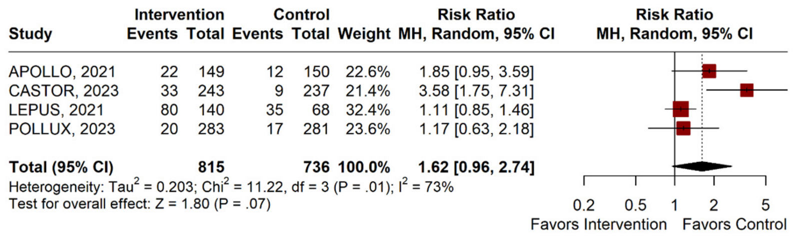

O.Nausea[43,46,47].

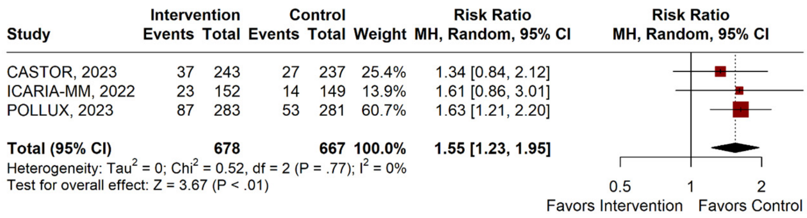

P. Neutropenia [42–46].

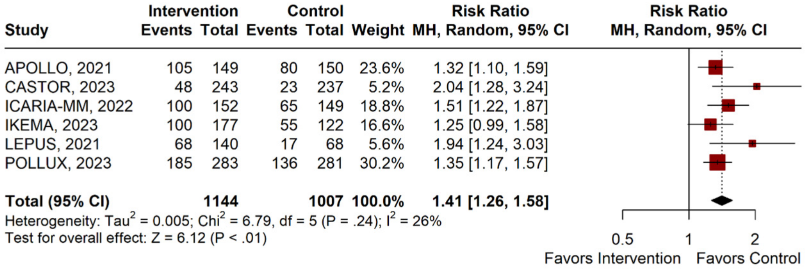

Q. Peripheral edema [42,46,47].

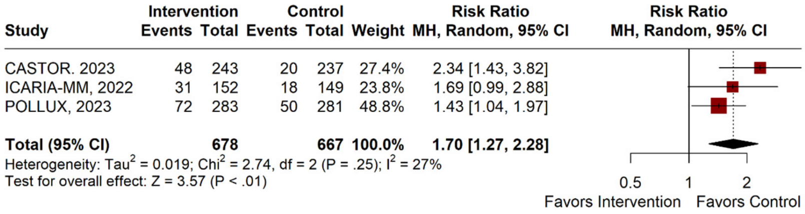

R. Pneumonia [42–47].

Study	Intervention Events	Intervention Total	Control Events	Control Total	Weight	Risk Ratio MH, Random, 95% CI
APOLLO, 2021	27	149	17	150	9.1%	1.60 [0.91, 2.81]
CASTOR, 2023	40	243	32	237	15.6%	1.22 [0.79, 1.87]
ICARIA-MM, 2022	51	152	44	149	25.7%	1.14 [0.81, 1.59]
IKEMA, 2023	48	177	26	122	16.5%	1.27 [0.84, 1.93]
LEPUS, 2021	17	140	7	68	4.2%	1.18 [0.51, 2.71]
POLLUX, 2023	80	283	49	281	29.0%	1.62 [1.18, 2.22]
**Total (95% CI)**		**1144**		**1007**	**100.0%**	**1.34 [1.13, 1.59]**

Heterogeneity: $Tau^2 = 0$; $Chi^2 = 3.06$, df = 5 (P = .69); $I^2 = 0\%$
Test for overall effect: Z = 3.39 (P < .01)

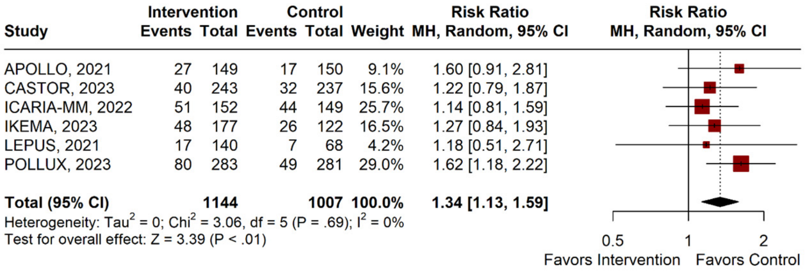

S. Pyrexia [42,43,45–47].

Study	Intervention Events	Intervention Total	Control Events	Control Total	Weight	Risk Ratio MH, Random, 95% CI
APOLLO, 2021	29	149	21	150	15.7%	1.39 [0.83, 2.32]
CASTOR, 2023	46	243	28	237	21.9%	1.60 [1.04, 2.47]
ICARIA-MM, 2022	27	152	21	149	15.1%	1.26 [0.75, 2.13]
LEPUS, 2021	43	140	11	68	11.7%	1.90 [1.05, 3.44]
POLLUX, 2023	77	283	41	281	35.6%	1.86 [1.33, 2.62]
**Total (95% CI)**		**967**		**885**	**100.0%**	**1.63 [1.33, 1.99]**

Heterogeneity: $Tau^2 = 0$; $Chi^2 = 2.15$, df = 4 (P = .71); $I^2 = 0\%$
Test for overall effect: Z = 4.69 (P < .01)

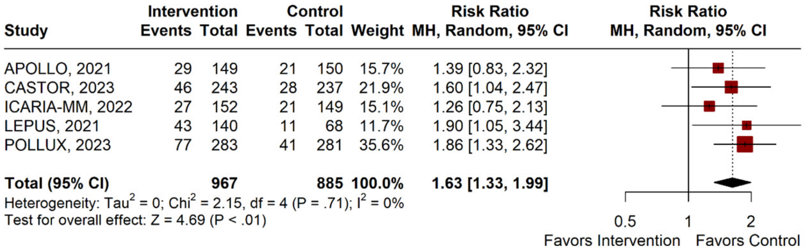

T. Thrombocytopenia [42–47].

Study	Intervention Events	Intervention Total	Control Events	Control Total	Weight	Risk Ratio MH, Random, 95% CI
APOLLO, 2021	48	149	49	150	8.5%	0.99 [0.71, 1.37]
CASTOR, 2023	145	243	105	237	19.1%	1.35 [1.13, 1.61]
ICARIA-MM, 2022	26	152	22	149	3.9%	1.16 [0.69, 1.95]
IKEMA, 2023	168	177	109	122	33.2%	1.06 [0.99, 1.14]
LEPUS, 2021	127	140	50	68	21.9%	1.23 [1.06, 1.44]
POLLUX, 2023	93	283	90	281	13.4%	1.03 [0.81, 1.30]
**Total (95% CI)**		**1144**		**1007**	**100.0%**	**1.14 [1.02, 1.27]**

Heterogeneity: $Tau^2 = 0.008$; $Chi^2 = 9.99$, df = 5 (P = .08); $I^2 = 50\%$
Test for overall effect: Z = 2.37 (P = .02)

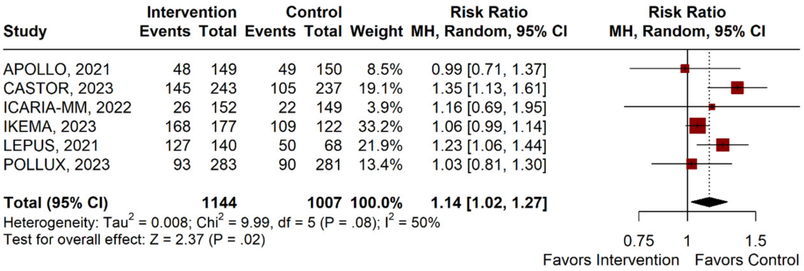

U. Upper respiratory tract infection [42–47].

Study	Intervention Events	Intervention Total	Control Events	Control Total	Weight	Risk Ratio MH, Random, 95% CI
APOLLO, 2021	34	149	24	150	8.5%	1.43 [0.89, 2.28]
CASTOR, 2023	90	243	43	237	18.9%	2.04 [1.49, 2.80]
ICARIA-MM, 2022	55	152	30	149	12.9%	1.80 [1.23, 2.64]
IKEMA, 2023	66	177	33	122	15.6%	1.38 [0.97, 1.95]
LEPUS, 2021	53	140	15	68	7.7%	1.72 [1.05, 2.81]
POLLUX, 2023	125	283	79	281	36.3%	1.57 [1.25, 1.97]
**Total (95% CI)**		**1144**		**1007**	**100.0%**	**1.64 [1.43, 1.89]**

Heterogeneity: $Tau^2 = 0$; $Chi^2 = 3.53$, df = 5 (P = .62); $I^2 = 0\%$
Test for overall effect: Z = 7.08 (P < .01)

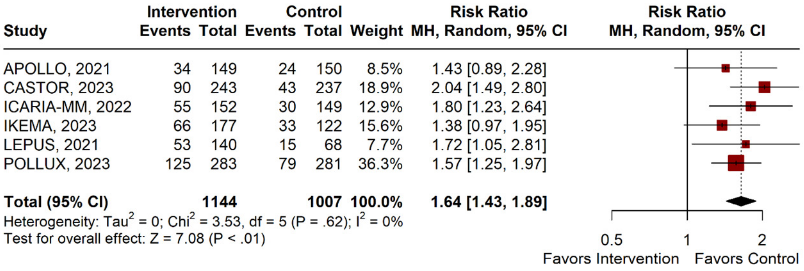

Supplementary Figure 2 Grade ≧3 of adverse events. **A.** Anemia. **B.** Arthralgia. **C.** Asthenia. **D.** Back pain**. E.** Bronchitis. **F.** Constipation**. G.** Cough**. H.** Diarrhea**. I.** Dyspnea. **J**. Fatigue. **K.** Febrile neutropenia. **L.** Hypertension. **M**.Insomnia. **N**.Lymphopenia. **O**.Nausea. **P.**Neutropenia**. Q**.Peripheral edema. **R**.Pneumonia. **S.** Pyrexia **T.** Thrombocytopenia. **U**. Upper respiratory tract infection [42–47].

A. Anemia [42–47].

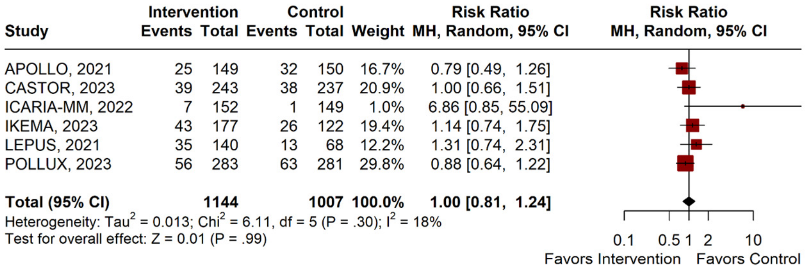

B. Arthralgia [43,44,46,47].

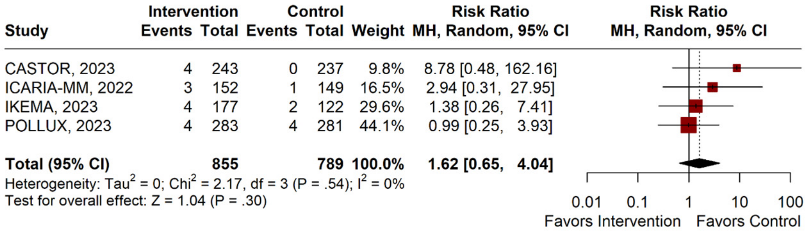

C. Asthenia [424–4,46,47].

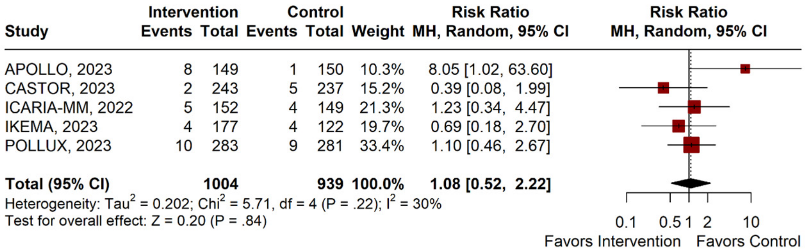

D. Back pain [43,44,46,47].

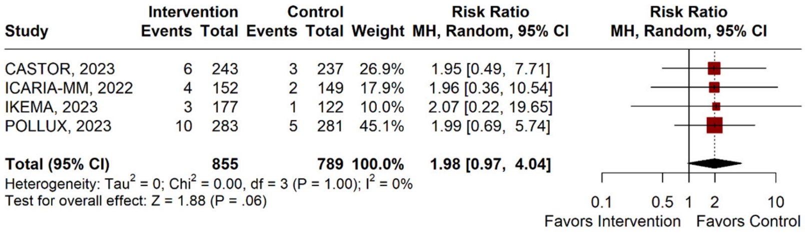

E. Bronchitis [43,44,46,47].

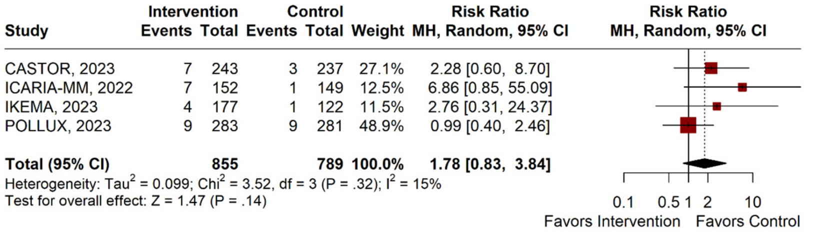

F. Constipation [43,45–47].

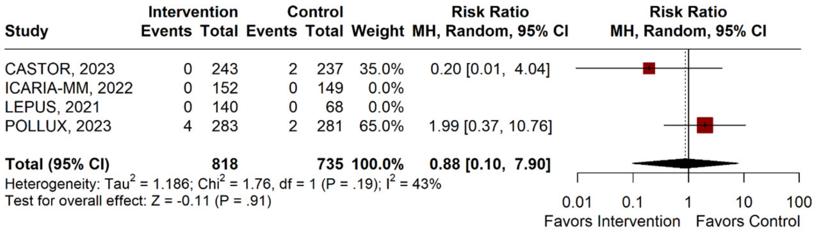

G. Cough [44–47].

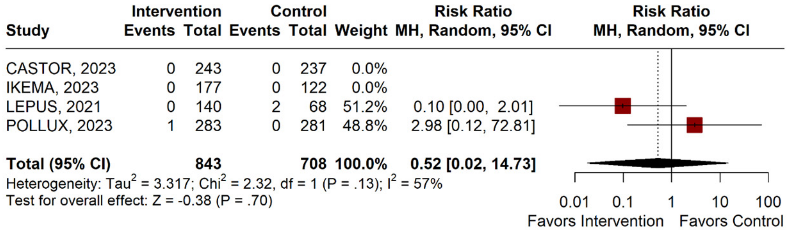

H. Diarrhea [42–47].

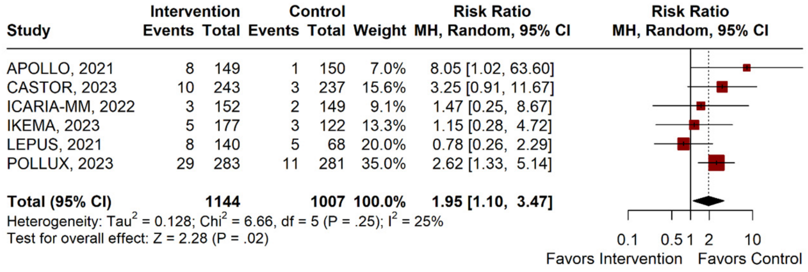

I. Dyspnea [43,44,46,47].

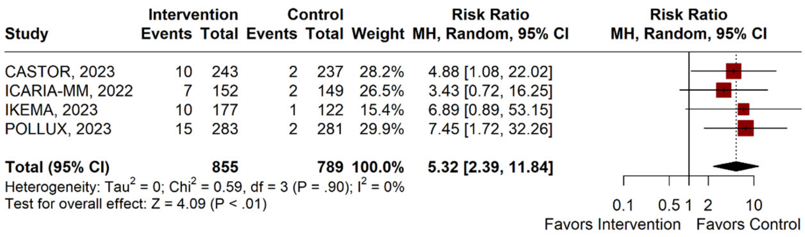

J. **Fatigue [42–44,46,47].**

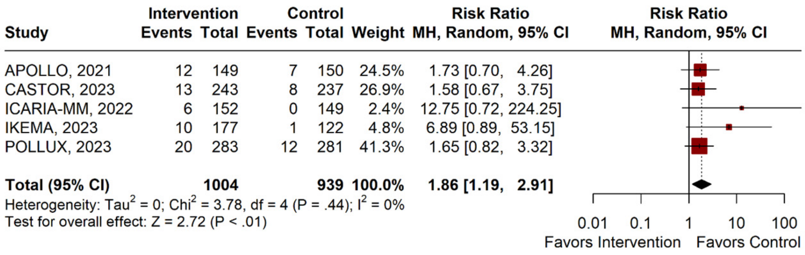

K. **Febrile neutropenia [42,43,47].**

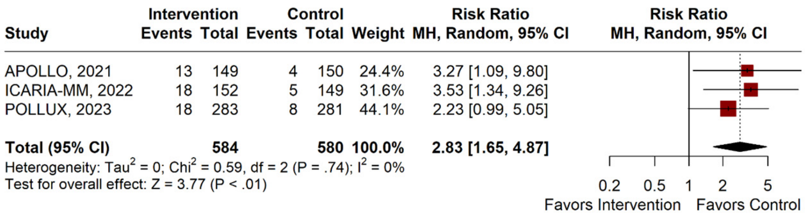

L. Hypertension [44,45,47].

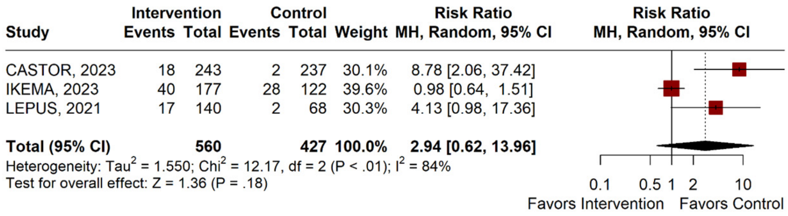

M. Insomnia [44–47].

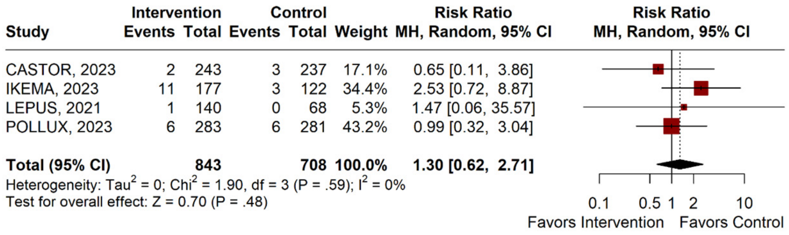

N. Lymphopenia [42,45–47].

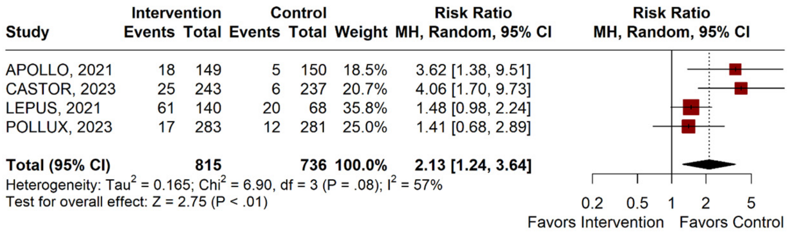

O. Nausea [43,46,47].

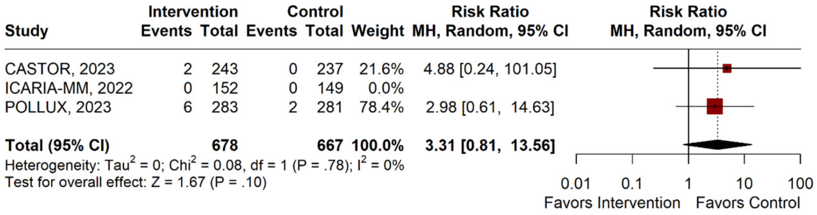

P. Neutropenia [42–47].

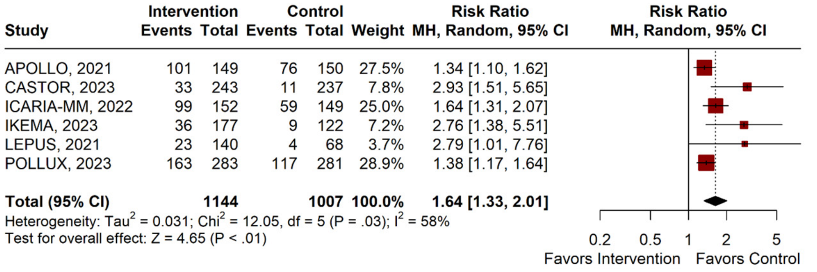

Q. Peripheral edema [43,46,47].

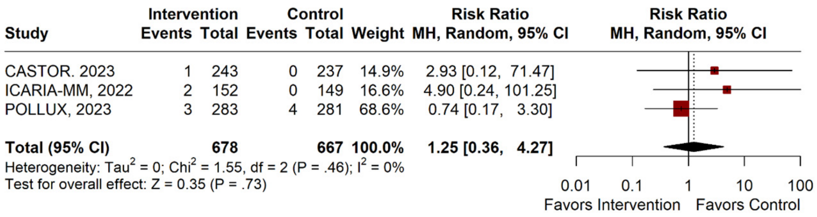

R. Pneumonia [42–47].

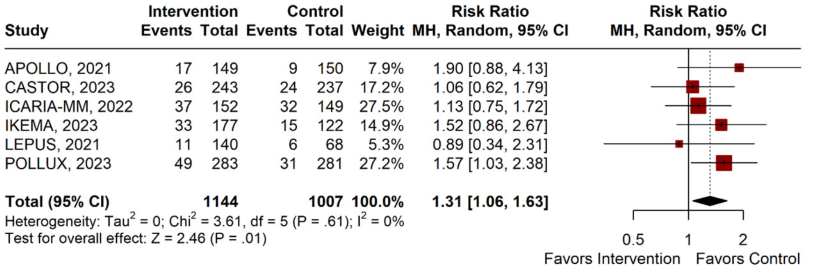

S.Pyrexia [42,43,45–47].

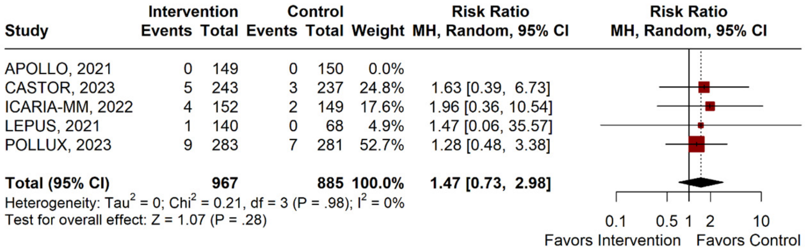

T. Thrombocytopenia [42–47].

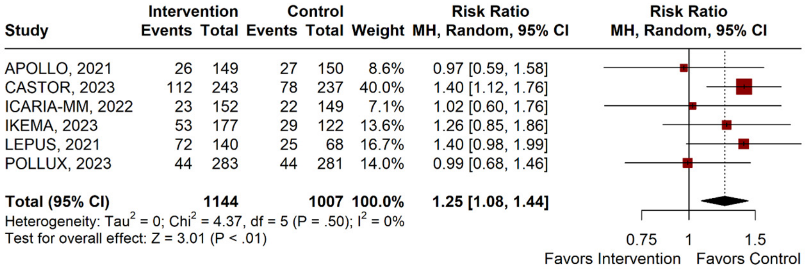

U. Upper respiratory tract infection [42–47].

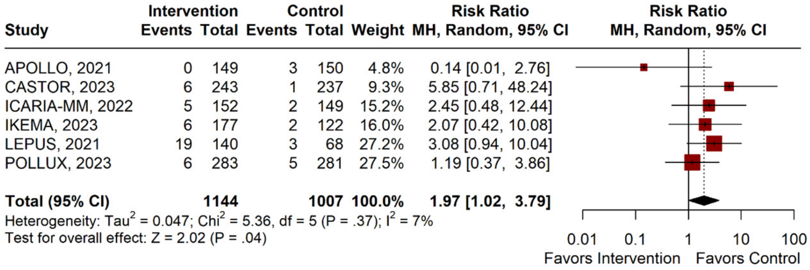

Supplementary Figure S3 Leave-one-out sensitivity analyses plots. **A.** Progression-free survival. **B.** Overall survival [42–47]**.**

A. Progression-free survival overall

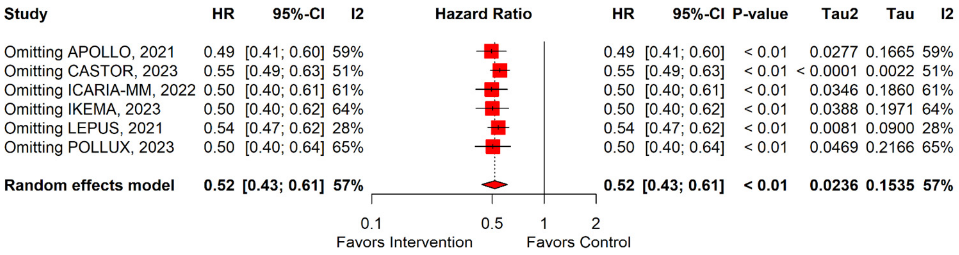

B. Overall survival

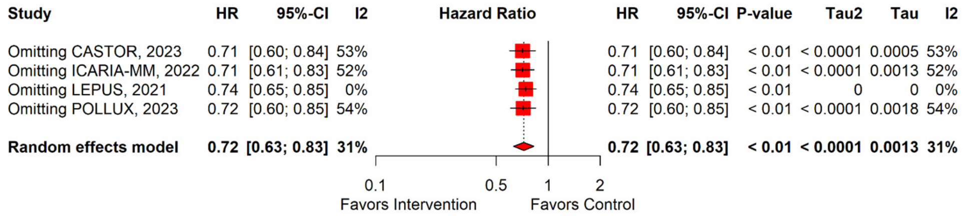

Supplementary Figure S4 Funnel plot of the overall survival outcome [43,45–47].

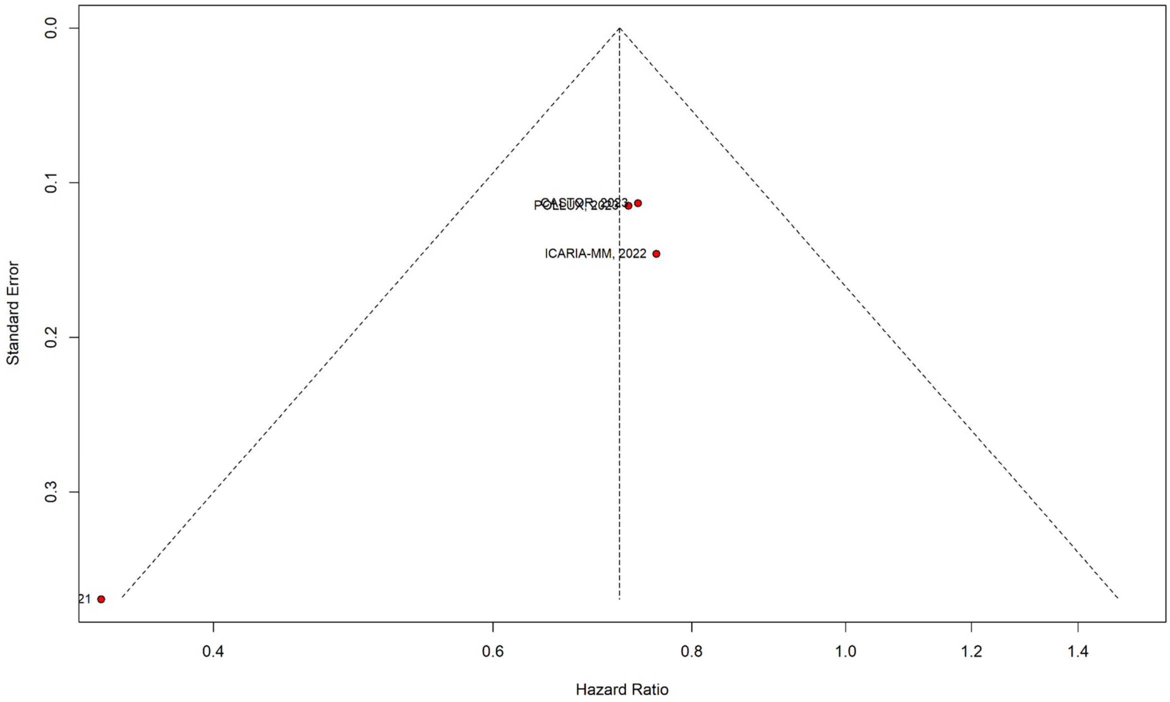

Supplementary Figure S5 Funnel plot of the progression-free survival outcome [42–47].

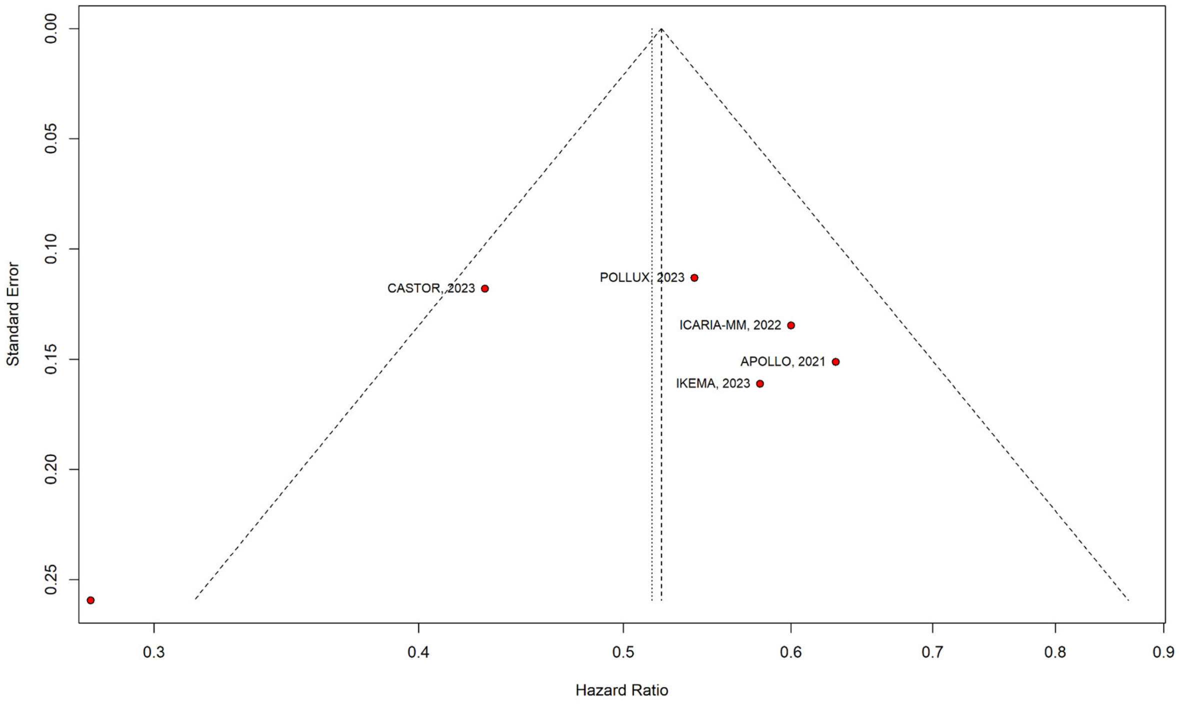

Supplementary Figure S6 Overall risk of bias [42–47].

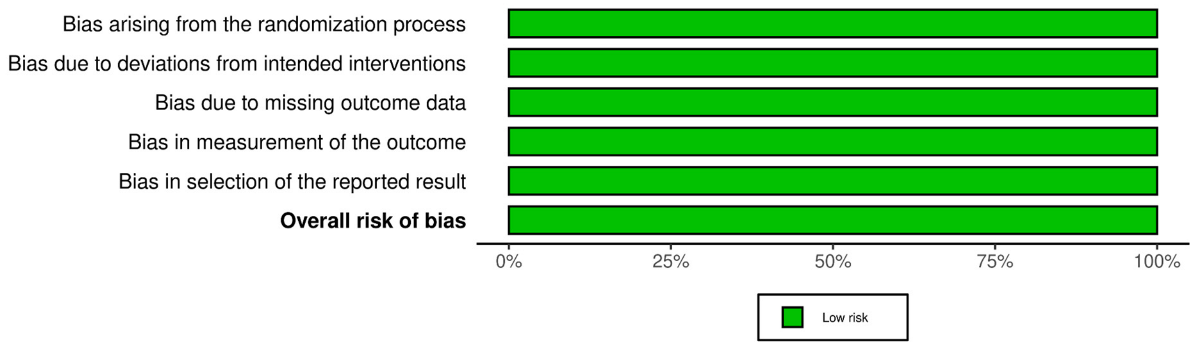

Supplement: Supplementary file 1 [file jpm-14-00360-s001.zip › jpm-2898144-supplementary.pdf]
